# Joint angle-diversity SDMA, OFDMA, and strict FFR framework for dense LiFi attocell networks

**DOI:** 10.1038/s41598-026-65724-w

**Published:** 2026-08-14

**Authors:** Mostafa Mohamed, Hosny M. Ibrahim, Ali H. Ahmed, Nagwa M. Omar

**Affiliations:** Information Technology Department, Faculty of Computers and Information, Assiut University, Assiut, 71515 Egypt

**Keywords:** LiFi, OFDMA, SDMA, Fractional frequency reuse, Angle-diversity transmitter, Mobility-aware proportional-fair scheduling, Engineering, Mathematics and computing

## Abstract

Dense deployment of LiFi and visible light communication attocells increases sensitivity to inter-cell interference, requiring coordinated interference and resource management. Angle-diversity transmitters (ADTs) for space-division multiple access (SDMA), OFDMA allocation, and fractional frequency reuse (FFR) have been studied separately under inconsistent assumptions, making it difficult to separate reuse-driven from activity- and resource-sharing effects. This work presents a protocol-level framework that unifies ADT-enabled SDMA, OFDMA, and strict FFR within an intensity-modulation direct-detection DCO-OFDM model. Each macrocell is served by a co-located multi-beam ADT defining beam-based microcells; users are classified as center or edge, and protected edge sub-bands are assigned using adjacency-aware graph coloring. Full-load and random-one-per-region regimes are evaluated in 10,000-trial Monte Carlo simulations with sensitivity sweeps over center-edge partitioning and user density. Both regimes achieve a mean SINR of about 28 dB, with no outage observed at 10 or 20 dB in the finite LOS sample. A dynamic extension incorporating mobility, receiver-orientation variation, a parametric first-order NLOS stress model, and variable multi-user OFDMA admission shows that OMN-PF improves edge-frame service success over conventional PF, while OMN-PF-A further improves high-threshold edge reliability with similar scheduled spectral efficiency. The framework provides a reproducible benchmark for dense LiFi resource management.

**Supplementary Information:**

The online version contains supplementary material available at 10.1038/s41598-026-65724-w.

## Introduction

LiFi and visible light communication (VLC) systems use the large unregulated optical spectrum and the widespread availability of solid-state lighting to deliver high-capacity indoor wireless links. Dense optical attocell deployment is attractive because it enables aggressive spatial reuse, but that same reuse can move the operating regime from noise-limited to interference-limited. In multi-attocell layouts, the dominant impairment is often inter-cell interference (ICI) created by overlapping illumination footprints, boundary users, and uncoordinated reuse among neighboring access points. Recent reviews of LiFi networking and optical wireless communication for 6G also show that practical indoor systems must deal with interference together with mobility, load balancing, and resource allocation^1,2^. Indoor LiFi networking studies and broader analyses of multi-user optical wireless interference reach the same conclusion: dense optical networking needs interference-aware cell planning, handover support, and technology-specific interference models^3,4^.

Several complementary directions have been explored to mitigate ICI in optical cellular networks. On the transmitter side, space-division multiple access (SDMA) using angle-diversity transmitters (ADTs) shapes optical radiation into narrower beams, improving spatial selectivity and reducing leakage power toward unintended users. The ADT concept and SDMA operation in optical attocells have been demonstrated as an effective interference mitigation mechanism, particularly when multiple narrow beams are used to serve spatially separated users in parallel^5^.

On the frequency side, fractional frequency reuse (FFR) has been adopted from RF cellular systems to reduce edge interference while preserving center reuse. In optical attocell networks, strict or soft reuse policies can partition OFDM data subcarriers into center and edge groups and allocate protected edge resources to limit co-channel interference among adjacent edge regions. FFR designs for DCO-OFDM-based optical attocells and their performance trade-offs have been studied in detail, including strict partitioning and related variants^6,7^.

OFDMA provides an additional degree of freedom by allocating disjoint subcarrier sets to multiple users within a cell, enabling fine-grained scheduling and load adaptation. Resource allocation and interference management for OFDMA-based VLC networks have been investigated, including strategies for subcarrier assignment, power distribution, and handover behavior in multi-cell scenarios. These studies highlight that system-level conclusions depend strongly on how OFDMA subcarriers are partitioned among users and on the assumed interference coupling across cells^8–10^. A recent OFDMA-VLC study further shows that BER and throughput are strongly affected by joint subcarrier and power-allocation choices, reinforcing the importance of reproducible OFDMA assumptions in system-level evaluations^11^. Additional indoor OFDMA-VLC resource-allocation algorithms and FOV-limited interference-avoidance analyses likewise confirm that allocation policy and cell-overlap handling strongly influence throughput and interference^12,13^.

Receiver-side mitigation has also received significant attention. Angle-diversity receivers (ADRs) and field-of-view (FOV) control can suppress interference by spatial filtering at the photodetector front end, effectively rejecting components arriving outside the receiver’s acceptance cone. ADR-based designs and optimized FOV selection have been shown to reduce ICI and improve robustness in LiFi cellular networks, particularly in dense deployments where interference dominates^14–16^. Recent dynamic-FoV and variable-focus receiver studies also show that adaptive receiver optics can provide additional interference suppression beyond fixed-FOV designs^17,18^. Related ADR-based interference-mitigation designs and dynamically adaptive wide-FoV receivers further support the value of receiver-side optical filtering in dense VLC networks^19,20^. Recent liquid–crystal and liquid-lens receiver studies further indicate that tunable optical front ends can support interference suppression and robustness under orientation and mobility variations^21,22^.

Prior studies provide the constituent mechanisms but answer different questions. Reference^5^ evaluates ADT-enabled SDMA, references^6,7^ study optical-cell FFR, and references^8–13^ address OFDMA resource allocation or interference management without reproducing the complete benchmark used here. This paper does not claim priority for any component or for graph coloring. Its narrower contribution is a common simulator with explicit interferer sets, shared IM/DD assumptions, two loading regimes, matched internal ablations, and a dynamic scheduler extension.

Against that background, this paper studies a protocol-level interference-control framework that combines ADT-based SDMA, OFDMA, and strict FFR under an IM/DD-consistent DCO-OFDM model. Each macrocell is served by one physical access point equipped with a 7-element co-located ADT. The seven microcells are beam-defined floor regions rather than physically separate access points. Users are classified through a center-radius fraction δ, and strict FFR assigns protected edge sub-bands through an adjacency-aware coloring rule so that adjacent edge regions do not reuse the same resources.

Two static loading regimes are evaluated. Under full load, all dropped users are active. Under random-one-per-region (rr1), one user is selected uniformly in each region in every independent trial and all users are redropped in the next trial. rr1 is therefore a scheduled-link sampling regime, not temporal round robin over persistent user identities.

The contribution of this work is therefore not the introduction of a new standalone physical-layer mechanism. ADT-enabled SDMA, OFDMA-based allocation, FFR reuse control, and receiver-side filtering are established in the LiFi/VLC literature, but they are often evaluated under different modeling assumptions, interferer definitions, loading regimes, and PHY abstractions. The methodological contribution here is to place these ingredients inside one internally consistent protocol-level benchmark: beam-defined ADT microcells, explicit center-user and edge-user interferer sets, strict-FFR edge-pool assignment, IM/DD-consistent DCO-OFDM signaling, and two clearly defined interference-loading regimes. This benchmark is intended to clarify which observed gains arise from spatial/angular selectivity, which arise from frequency-reuse protection, and which arise from scheduler loading. The results should therefore be interpreted as controlled evidence for the evaluated static baseline and dynamic stress-extension settings, rather than as a claim of general optimality or universal deployment performance.

### Main contributions

- We formulate a joint ADT-enabled SDMA and OFDMA framework with strict FFR for dense optical attocells, explicitly defining center/edge classification, subcarrier pools, and the co-channel interferer sets used in SINR evaluation.
- We model a co-located 7-beam ADT macrocell in which microcells are beam-defined floor regions, and we implement a standard adjacency-aware greedy graph-coloring rule for protected edge-pool assignment, as described in “Edge-pool graph coloring implementation” Section, while reporting the resulting conflict count.
- We use a Hermitian-symmetric QPSK DCO-OFDM/OFDMA waveform with PSD-normalized thermal noise. The implemented dynamic bias makes the waveform non-negative, so residual clipping noise is zero in the reported static runs; LED nonlinearities and finite headroom are not modeled.
- We evaluate full-load and random-one-per-region (rr1) static regimes to separate simultaneous resource sharing from scheduled-link sampling, and we sweep the center/edge partition ratio δ and offered user density.
- We provide a reproducible Monte Carlo workflow (Algorithms 1–2) that exports the CSV summaries from which the reported figures and tables are produced. An ablation driver provides controlled internal variants that isolate the effect of ADT selectivity, OFDMA partitioning, and strict-FFR edge protection; these variants are used for the quantitative comparison reported in Table 5 and are not intended as exact re-implementations of individual prior studies. In addition, a dynamic extension evaluates mobility, time-correlated receiver orientation, first-order NLOS stress, beam-transition risk, variable multi-user OFDMA admission, service-gap-aware OMN-PF scheduling, and the OMN-PF-A adaptive OFDMA/power operating mode.

The remainder of this paper is organized as follows. “Related work” Section reviews the related literature. “System model” Section presents the system model and LOS channel formulation. “Proposed framework” Section details the proposed ADT-enabled SDMA, OFDMA, and strict-FFR framework. “Evaluation metrics” Section defines the evaluation metrics. “Experiments and Monte Carlo methodology” Section describes the Monte Carlo methodology and simulation assumptions. “Results and discussion” Section discusses the static numerical results, “Comparison with related work” Section compares the proposed design with representative related work, “Dynamic reliability-aware extension under mobility and NLOS stress” Section adds a dynamic reliability-aware multi-user OFDMA extension under mobility and NLOS stress, “Limitations and scope of validity” Section summarizes the limitations and scope of validity, and “Conclusion” Section concludes the paper.

## Related work

Interference management in optical attocell networks has largely progressed along separate lines rather than within one shared evaluation framework. Some studies emphasize transmitter-side spatial selectivity through ADT-enabled SDMA or receiver-side angular filtering, others focus on OFDMA allocation rules, and others study FFR reuse patterns in DCO-OFDM optical cells. As a result, reported gains are often evaluated under different modeling assumptions, PHY abstractions, and interference conditions. The purpose of the present related-work discussion is therefore not only to summarize these strands, but also to show where the modeling gap lies: the literature contains the ingredients, but much less often a unified framework that evaluates them together under explicit interferer sets, shared IM/DD assumptions, and scheduler-aware interference regimes^3,4,11–13,17–20^.

SDMA exploits the spatial domain to serve multiple users simultaneously using distinct directional beams. In optical attocell networks, ADTs implement SDMA by generating parallel narrow beams, improving spatial selectivity and reducing leakage toward unintended receivers^5^.

OFDMA divides the available bandwidth into orthogonal subcarriers and allocates disjoint subcarrier sets to users, enabling flexible multiuser scheduling and load adaptation. Resource-allocation studies in multi-cell VLC highlight that interference coupling depends on subcarrier partitioning and activity assumptions^8–10^. Recent analysis of OFDMA VLC systems also confirms the sensitivity of BER and achievable rate to the adopted allocation policy^11^. Complementary indoor OFDMA-VLC allocation algorithms and FOV-limited resource-reallocation studies further show that interference-aware allocation remains a central design lever in dense VLC networks^12,13^.

FFR mitigates edge interference by partitioning subcarriers into center and edge pools, allowing full reuse in the center while assigning protected edge resources to adjacent cells. In DCO-OFDM optical attocells, strict and soft FFR variants quantify the trade-off between edge protection and spectrum utilization^6,7^. Complementary receiver-side approaches, including dynamic-FoV and variable-focus optical receivers, have recently been explored as additional means of interference suppression in dense LiFi/VLC deployments^17,18^. Additional ADR-based and adaptive wide-FoV receiver studies reinforce the same conclusion from different deployment perspectives^19,20^.

Cross-domain cooperative compressed-spectrum-sensing studies provide a useful conceptual comparison. Enhanced and new cooperative schemes exploit spatial diversity, virtual data synthesis, and multi-user averaging to improve sparse recovery under low-SNR conditions^23,24^, while blind weighted fusion and overlapping-subband selection adapt the fusion process to variations in channel quality^25^. A related multivector reduced-set matching pursuit method reduces the computational burden of joint sparse recovery by sharing support estimation across multiple measurement vectors and pruning the search process^26^. These studies address RF spectrum sensing and information fusion rather than LiFi transmission; nevertheless, they illustrate the broader coordination problem of making fusion or resource decisions when participating links have heterogeneous quality. The parallel is conceptual, and no algorithmic equivalence with the OFDMA and strict-FFR framework is implied.

## System model

### Network layout and ADT geometry

Macrocells are arranged on a hexagonal grid (multi-ring layout). Each macrocell contains a single co-located ADT at the macrocell center. The seven microcells are logical floor regions associated with the ADT beams (one central region and six surrounding regions) and are used for user placement and adjacency; the physical transmitter location is identical for all beams. Beam selection applies an angular acceptance constraint (beam half-angle) relative to each beam axis.

### User distribution and center/edge classification

**Fig. 1:**
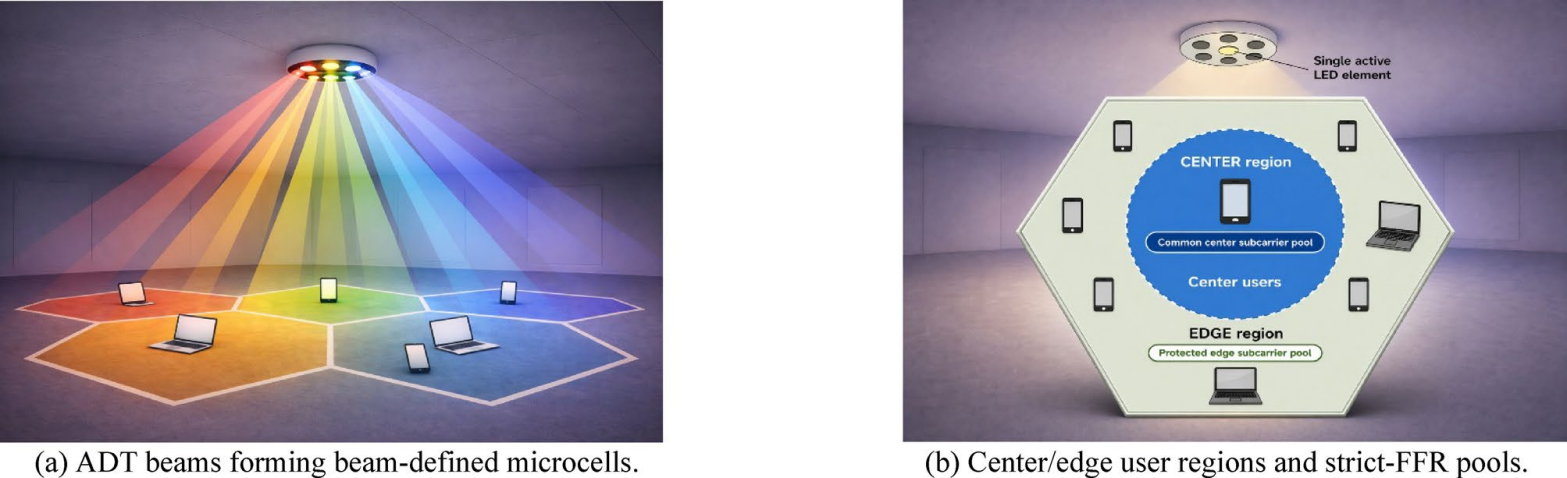
Conceptual illustration of the static framework: (**a**) co-located ADT beams forming beam-defined regions; (**b**) the center/edge classification applied within a representative region and the strict-FFR pools. Panel (**b**) is schematic and does not depict the three edge-pool colors.

Users are dropped uniformly within each beam-defined region of radius R. Center/edge classification uses *R*_*c*_ = δR. The static full-load regime activates all dropped users; rr1 selects one uniformly random user per region per independent trial. Because identities do not persist across trials, rr1 does not measure long-term round-robin service (Fig. 1).

### OFDMA and strict-FFR resource partitioning

Within each microcell, users are allocated disjoint OFDMA data subcarriers. The usable DCO-OFDM data subcarriers have cardinality *N*_*data*_ = *K*/2 − 1. A strict-FFR policy partitions these data subcarriers into a common center pool and three protected edge pools. The center pool size is *N*_*c*_ = ⌊δ^2^*N*_*data*_⌋ and the remaining subcarriers are divided equally among the three edge pools. Adjacent edge microcells are assigned different edge pools using an adjacency-aware coloring rule to limit edge co-channel interference while preserving full reuse in the center pool.

The parameter δ is therefore a design and evaluation parameter rather than a universally optimized constant. It simultaneously controls the physical center-region radius and the OFDMA partition between the fully reused center pool and the protected edge pools. Increasing δ enlarges the center region and increases the number of center-pool subcarriers, which can improve reuse efficiency but may expose a larger fraction of boundary users to center-pool interference. Decreasing δ moves more users into the edge class and increases protection, but it reduces the fully reused center region and can lower spectral-efficiency potential. For this reason, the baseline value δ = 2/3 is used as a balanced reference, and “Sensitivity study (scheduler regimes)” Section reports a sensitivity sweep over δ *∈* {0.5, 2/3, 0.8} rather than claiming global optimality.

### Assumptions

Unless stated otherwise, the static baseline experiments use the following controlled assumptions: (i) LOS-dominant optical propagation, with NLOS reflections and blockage excluded from the baseline channel; (ii) static user snapshots in the main benchmark, with frame-based mobility and serving-region transitions introduced later in “Dynamic reliability-aware extension under mobility and NLOS stress” Section; (iii) upward-facing receivers with fixed orientation in the baseline, relaxed in “Orientation sensitivity” and “Dynamic reliability-aware extension under mobility and NLOS stress” Sections; (iv) FOV filtering represented at the protocol level; and (v) equal power across active OFDMA data subcarriers in the static benchmark. These assumptions isolate the effect of ADT beam selectivity and strict-FFR resource partitioning. “Dynamic reliability-aware extension under mobility and NLOS stress” Section retains the validated fixed partition δ = 2/3, but adds variable multi-user OFDMA admission and compares equal-resource OMN-PF with the normalized adaptive OFDMA/power mode OMN-PF-A.

The simulator instantiates this model with a single co-located seven-element ADT per macrocell, PSD-normalized thermal-noise variance, and the OFDMA/FFR partition defined above; implementation-specific parameter values are listed in “Experiments and Monte Carlo methodology” Section.

The 3 W value in Table 1 is a controlled electrical-power input to the simulator, not an illumination-grade or universally optimal operating point^27^. With *η*_LED_ = 0.30 it gives 0.90 W optical power per macrocell; the implemented beam split assigns 0.36 W to the central beam and 0.09 W to each of the six outer beams. For K = 512, *N*_data_ = 255, B = 5 MHz, and *N*_*0*_ = 10^–21^ A2/Hz, the modeled per-subcarrier thermal-noise variance is *N*_*s*_ = *N*_*0*_B/*N*_data_ = 1.96 × 10^–17^ A2. Desired and interfering electrical terms scale quadratically with allocated optical power, whereas this noise term does not; the results are therefore not assumed invariant to transmit-power scaling.

**Table 1:** Simulation parameters used in the Monte Carlo evaluation.

Parameter	Description	Value
Geometry and layout		
Layout	Hexagonal macrocell scope	2 rings; 19 macrocells; 7 co-located logical regions/macrocell
* h*_*f*_	Transmitter–receiver height difference	2.15 m
R	Logical-region radius	3.3 m
* R*_*c*_	Center radius	2.2 m
δ	Center-radius fraction	2/3
Optical transmitter and receiver		
* A*_*r*_	Photodiode active area	5.0 × 10^−5^ m^2^ (0.5 cm^2^)
* T*_*s*_	Optical-filter transmission	0.95
n	Concentrator refractive index	1.5
* Ψ*_*c*_	Static receiver FOV semi-angle	40°
Φ_1/2_	LED half-power semi-angle	60°
m	Lambertian order	1
η	Photodiode responsivity	0.40 A/W
* N*_LED_	Co-located beam axes	7
Beam spacing	Static azimuth-axis spacing	360°/7 = 51.43°
Beam gate	Static beam-selection half-angle	20°
OFDMA and strict FFR		
K/*N*_data_	IFFT size/usable positive-frequency tones	512/255
* N*_*c*_/*N*_*e*_	Center pool/each protected edge pool	113/47
* B*_*e*_	Protected edge pools	3
Reserved tone	Trailing usable tone outside equal pools	1
B	System bandwidth	5 MHz
Noise and power		
* N*_0_	Thermal-noise PSD	10^−21^ A^2^/Hz
* N*_*s*_	Thermal-noise variance per data tone	1.96 × 10^−17^ A^2^
* P*_*t,electrical*_	Electrical input per macrocell	3 W
* η*_LED_	Electro-optical efficiency	0.30
* P*_*optical,macro*_	Optical macrocell budget	0.90 W
* P*_center_/*P*_outer_	Static beam budgets	0.36 W/0.09 W per outer beam
Power-control exponent	Optional distance-control exponent	0 (disabled)
* s*_DC_	Dynamic per-symbol bias	− min_*n*_ x[n]
Monte Carlo		
Trials/users	Per configuration	10,000/42 users per macrocell
Static regimes	Active-link sampling	full-load; random-one-per-region (rr1)

All static variants are compared at the same 3 W input and the numerical values are treated as configuration-specific benchmark estimates. The simulation does not contain the spectral, radiance, exposure, modulation-depth, illuminance, or hardware data needed to establish compliance. A deployment would require separate illumination, photobiological-safety, flicker, LED-nonlinearity, and bias/power-budget assessments under applicable standards such as IEC 62471 and IEEE Std 1789^28,29^.

### Line-of-sight propagation model

**Fig. 2:**
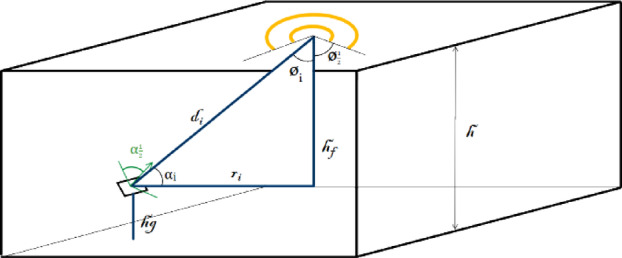
LOS optical channel geometry showing distance and angular terms.

A line-of-sight (LOS) optical link is considered between the ceiling-mounted access point and a user device. Let *r*_*i*_ denote the horizontal separation and *h*_*f*_ the transmitter–receiver height difference, so the propagation distance is *d*_*i*_ = √(*r*_*i*_2 + *h*_*f*_2). The irradiance angle *φ*_*i*_ is measured with respect to the transmitter axis, and the incidence angle *ψ*_*i*_ is measured with respect to the receiver axis,  as illustrated in Fig. 2.

Under Lambertian emission and IM/DD reception, following the standard indoor optical wireless channel formulation^30^, the LOS DC channel gain *G*_*i*_(*r*_*i*_) is given by (1) for 0 ≤ *ψ*_*i*_ ≤ *Ψ*_*c*_ and is zero otherwise. In (1), *A*_*r*_ is the photodiode active area, *T*_*s*_(*ψ*_*i*_) is the optical filter transmission, and *g*(*ψ*_*i*_) is the optical concentrator gain.

The Lambertian order *m* is determined by the LED half-power semi-angle *Φ*_1/2_ as in (2). The concentrator gain *g*(*ψ*) follows (3), where *n* is the refractive index of the concentrator and *Ψ*_*c*_ is the receiver field-of-view (FOV) semi-angle.

1 $$G_{i} \left( {r_{i} } \right) = \frac{{\left( {m + 1} \right)A_{r} }}{{2\pi d_{i}^{2} }}{\mathrm{cos}}\left( {\psi_{i} } \right)g\left( {\psi_{i} } \right){\mathrm{cos}}^{m} \left( {\phi_{i} } \right)T_{s} \left( {\psi_{i} } \right),\quad 0 \le \psi_{i} \le \Psi_{c}$$

2 $$m = - \frac{{{\mathrm{ln}}2}}{{{\mathrm{ln}}\left( {{\mathrm{cos}}\Phi_{1/2} } \right)}}$$

3 $$g\left( \psi \right) = \frac{{n^{2} }}{{{\mathrm{sin}}^{2} \left( {\Psi_{c} } \right)}},\quad 0 \le \psi \le \Psi_{c}$$

In addition, the per-subcarrier noise term used in subsequent SINR calculations is *N*_*s*_ = *N*_*0*_* B*/*N*_*data*_, where *N*_*data*_ = *K*/2 − 1.

### Horizontal distance calculation

**Fig. 3:**
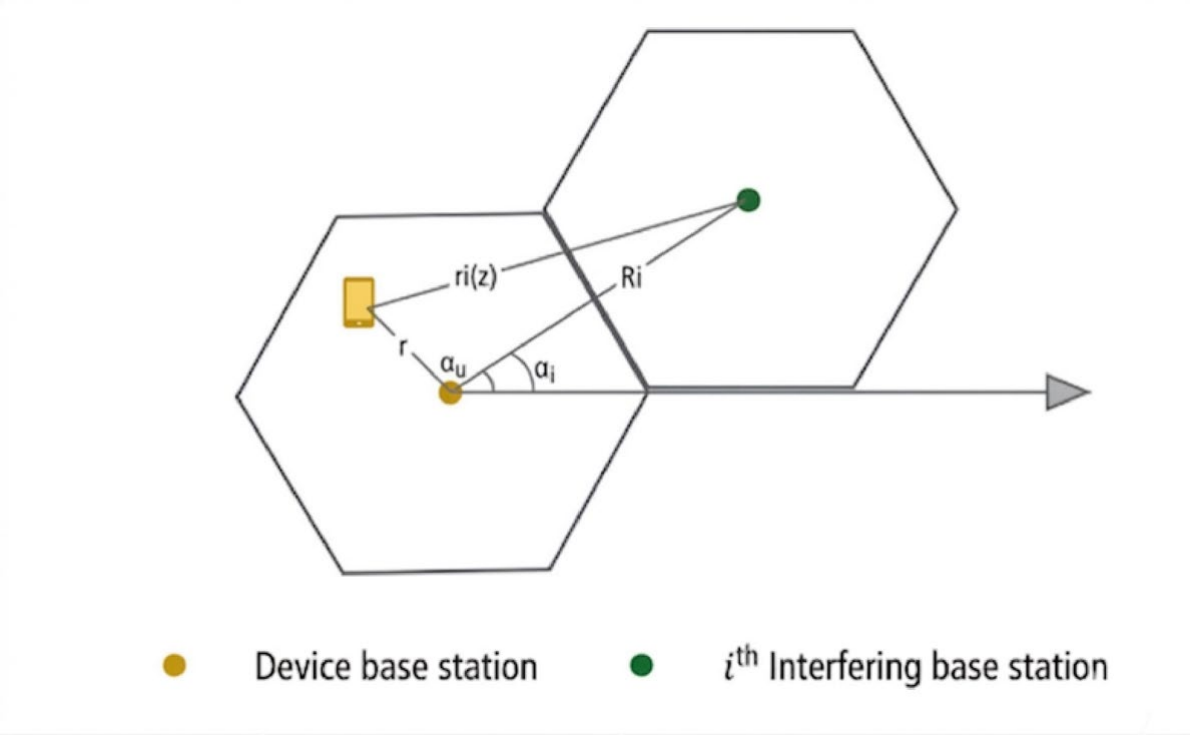
Horizontal-distance geometry for serving and interfering base stations.

To determine the horizontal distance between the user device and interfering base stations in our LiFi system, we use the following equation, adapted from^7^ (Fig. 3):

4 $$r_{i} \left( z \right) = \sqrt {\left( {r^{2} + R_{i}^{2} - 2rR_{i} cos\left( {\alpha_{u} - \alpha_{i} } \right)} \right)}$$

Here, *r*_*i*_(*z*) denotes the horizontal distance between the *i*-th base station and the user device positioned at *z*, where *z* = (*r*, *α*_*u*_). The variable *r* represents the distance from the user device to the center of the hexagonal cell, while *R*_*i*_ represents the distance between the *i*-th base station and the serving base station. The polar angle of the user device is denoted by *α*_*u*_, while *α*_*i*_ represents the polar angle of the *i*-th base station. This equation captures the geometric layout of the hexagonal cell structure and accounts for the angular positions of the user device and base stations. It therefore enables systematic computation of the horizontal distances to all interfering base stations from the user device within the central cell, supporting the overall interference analysis of the LiFi system.

### Scope of LOS-only baseline and dynamic extension

The main benchmark deliberately uses a static LOS-dominant setting. This choice isolates the interference-control effects of ADT angular selectivity, OFDMA subcarrier partitioning, and strict-FFR edge protection under a controlled and reproducible protocol-level model. Therefore, the static results should be read as baseline interference-control results rather than as claims for all indoor LiFi deployments. Practical indoor LiFi channels may include reflected optical components, human blockage, furniture shadowing, and mobility-induced changes in beam association. To address this limitation without changing the role of the baseline framework, “Dynamic reliability-aware extension under mobility and NLOS stress” Section adds a dynamic reliability-aware extension that introduces frame-based mobility, time-varying receiver orientation, first-order NLOS stress, serving-region transitions, and service-gap-aware PF scheduling.

In practical indoor LiFi systems, diffuse and reflected optical components may contribute additional received power and may also introduce additional inter-cell interference depending on room geometry, wall reflectivity, receiver field-of-view, and user orientation^3,4,30–33^. Similarly, user mobility can change the serving-beam association, center/edge status, and dominant interferer set over time. The dynamic extension in “Dynamic reliability-aware extension under mobility and NLOS stress” Section therefore evaluates these effects at the network-simulation level while preserving the ADT-SDMA-OFDMA strict-FFR resource structure introduced in the static benchmark.

Prior indoor optical-channel studies show that diffuse and reflected NLOS components can contribute a non-negligible fraction of the received optical power, depending on room geometry, surface reflectivity, receiver FOV, and user orientation; these components may add useful received power while also increasing inter-cell interference^30–36^. Consequently, the LOS-static baseline may overestimate the isolation produced by FOV filtering and ADT angular selectivity, while the “Dynamic reliability-aware extension under mobility and NLOS stress” Section NLOS-stress model should be interpreted as a bounded sensitivity test rather than a calibrated ray-tracing replacement.

The dynamic extension implements this idea by recomputing user position, receiver orientation, serving beam, center/edge status, interference set, and strict-FFR resource pool at each frame. It also adds a first-order NLOS-stress term, a handover-aware transition-risk state, variable resource- and SINR-aware multi-user admission, and two OMN-PF resource-allocation modes. Full deterministic room-scale ray tracing, measured blockage traces, hardware-level LED/receiver impairments, and lower-layer handover signaling remain outside the model, as discussed explicitly in “Limitations and scope of validity” Section.

## Proposed framework

The proposed framework implements the system model as a protocol-level interference-control scheme. It combines ADT-enabled spatial separation, OFDMA subcarrier allocation, and strict FFR edge protection, then defines the SINR expressions used to evaluate center and edge users.

### Framework overview

At the protocol level, each macrocell is served by a single co-located seven-element ADT. The seven ADT beams define logical microcell regions on the floor. Users in these regions share a common center subcarrier pool when they are classified as center users, while edge users are assigned protected subcarrier pools according to the strict-FFR rule. This structure allows spatial reuse through directional ADT beams while limiting co-channel interference among neighboring edge regions.

#### Edge-pool graph coloring implementation

The strict-FFR edge-pool assignment is implemented as a standard adjacency-aware greedy coloring of the edge-region adjacency graph, rather than as a new graph-theoretic optimization method. Each edge microcell is represented as a graph node. An undirected edge is inserted between two nodes when the corresponding edge regions are adjacent and may therefore create strong mutual edge interference if they reuse the same protected pool. The number of available edge colors is *B*_*e*_ = 3, corresponding to the three protected edge sub-bands used in the baseline configuration.

The use of three protected edge pools follows from the adopted seven-region adjacency topology. Let v0 denote the central beam-defined microcell and v1, …, v6 the six surrounding microcells. The central region and any two consecutive surrounding regions form a triangle in the edge-region adjacency graph, so at least three colors are required. Conversely, assigning one protected pool to the central region and alternating the other two pools around the six-region cycle gives a conflict-free three-color construction. Hence, for the considered topology, the minimum protected-pool count is *B*_*e,min*_ = χ(*G*_*e*_) = 3. The coordinated assignment is extended across the multi-ring layout and checked by the simulator’s adjacent same-pool conflict count.

The implementation applies a largest-degree-first greedy coloring to the constructed edge-region graph. The resulting color index is mapped to one of the three edge sub-bands, and the simulator records a conflict count after coloring. A conflict is counted when an adjacent pair of edge regions is assigned the same protected edge sub-band. In the reported baseline and sensitivity simulations, the recorded conflict count is zero, indicating that adjacent edge regions do not reuse the same protected edge pool under the considered hexagonal layout.

This coloring rule is used as an implementation mechanism for strict-FFR edge protection. It should not be interpreted as a novel graph-coloring algorithm; its role is to make the edge-pool assignment explicit, reproducible, and consistent across the Monte Carlo experiments.

More broadly, cooperative systems may involve a trade-off between coordination quality and receiver-side processing complexity. The multivector reduced-set matching pursuit (MRMP) method provides a cross-domain example by reducing the computational burden of joint sparse recovery through shared support estimation across multiple measurement vectors and a pruned search process^26^. The present framework does not employ sparse recovery; its OFDMA and strict-FFR partitioning is determined by interference topology and fixed resource constraints using a standard greedy coloring rule. The comparison is therefore conceptual, and MRMP is not used as the basis of the proposed graph-coloring or subcarrier-allocation procedure.

### SINR formulation

The SINR expressions evaluate the proposed resource-partitioning scheme for center and edge users. The desired and interfering channel gains follow the standard Lambertian LOS optical wireless channel formulation used for indoor optical links ^30^. Separate expressions are needed because the two groups reuse different subcarrier pools and therefore experience different co-channel interferer sets. Center users share the common center pool, whereas edge users are protected by strict-FFR edge pools that reduce adjacent-region interference.

#### Microcell SINR calculation

The radius of the center region is defined as *R*_*c*_ = δ*R*, where 0 < δ < 1, and the edge-region radius is *R*_*e*_ = *R* − *R*_*c*_. Given the usable data-subcarrier count *N*_*data*_, the center sub-band contains *N*_*c*_ subcarriers, while the remaining subcarriers are divided equally among the three edge sub-bands, giving *N*_*e*_ = (*N*_*data*_ − *N*_*c*_)/3. For a center user, the SINR is given by Eq. (5).

In Eq. (5), *η* is the photodiode responsivity, *S*_*u*_ is the set of subcarriers assigned to the center user, *P*_*t,s*_ and *P*_*t,j,s*_ denote the transmit power on subcarrier *s* for the desired user and the interfering base station *j*, respectively, *G*_*center,s*_ and *G*_*center,j,s*_ denote the desired and interfering channel gains, *I*_*center*_ is the set of interfering base stations using the same center sub-band, and *N*_*s*_ is the per-subcarrier noise power. If both the gain and the transmit power are assumed constant across the allocated subcarriers, the summation can be replaced by multiplying by the number of assigned subcarriers *N*_*sc,u*_.

5 $${\mathrm{SINR}}_{{{\mathrm{center}},{\mathrm{s}}}} = \frac{{\left( {\eta P_{{{\mathrm{t}},{\mathrm{s}}}} G_{{0,{\mathrm{s}}}} } \right)^{2} }}{{\mathop \sum \nolimits_{{j \in I_{{{\mathrm{center}}}} }} \left( {\eta P_{{{\mathrm{t}},{\mathrm{j}},{\mathrm{s}}}} G_{{{\mathrm{j}},{\mathrm{s}}}} } \right)^{2} + N_{s} }}$$

For an edge user, the SINR is given by Eq. (6).

6 $${\mathrm{SINR}}_{{{\mathrm{edge}},{\mathrm{s}}}} = \frac{{\left( {\eta P_{{{\mathrm{t}},{\mathrm{s}}}} G_{{0,{\mathrm{s}}}} } \right)^{2} }}{{\mathop \sum \nolimits_{{j \in I_{{{\mathrm{edge}}}} }} \left( {\eta P_{{{\mathrm{t}},{\mathrm{j}},{\mathrm{s}}}} G_{{{\mathrm{j}},{\mathrm{s}}}} } \right)^{2} + N_{s} }}$$

In Eq. (6), *I*_*edge*_ denotes the set of interfering base stations using the same protected edge sub-band. For center users, all nearby base stations reusing the common center pool contribute to the interference term, whereas for edge users only the neighboring base stations using the same protected edge sub-band contribute. The per-subcarrier noise power *N*_*s*_ is modeled using the PSD-based thermal-noise assumption and is taken to be the same for both center and edge users.

### DCO-OFDM-based optical attocell networks

The static simulator uses a DCO-OFDM signal model in which the total transmit power *P*_*t*_ is distributed uniformly across the active subcarriers. Under equal-power allocation, each active subcarrier carries *P*_*t,s*_ = *P*_*t*_/*N*_data_. The dynamic OMN-PF mode retains equal OFDMA sharing and uniform normalized regional power, whereas OMN-PF-A in “Dynamic reliability-aware extension under mobility and NLOS stress” Section applies bounded adaptive OFDMA shares and normalized adaptive power redistribution while preserving the same total regional power budget.

A DC bias s_DC_ is added so that the time-domain signal is non-negative for IM/DD transmission, and any remaining negative samples are clipped. Under strict FFR, center users draw from the common center pool, whereas edge users draw from protected pools that are not reused by adjacent edge regions.

#### Symbol generation in DCO-OFDM

The transmit chain is implemented in four steps: symbol mapping, Hermitian-symmetry enforcement, IDFT, and DC-bias/clipping.

Data modulation uses QPSK symbols placed on the positive-frequency data tones with Hermitian symmetry, which produces a real IFFT waveform x[n] under the DCO-OFDM construction^37,38^.

Hermitian symmetry is applied to ensure that the resulting OFDM symbols are real-valued, as required for optical intensity modulation. This symmetry is enforced by setting Sk = S*_K−k_ for k = 1, …, K/2 − 1, with S0 = *S*_*K*/2_ = 0, where * denotes the complex conjugate.

After symbol mapping and Hermitian symmetry, the IDFT converts the frequency-domain symbols into the real-valued time-domain OFDM signal:

7 $$s\left( t \right) = \frac{1}{K}\mathop \sum \limits_{k = 0}^{K - 1} S_{k} e^{j2\pi kt/K} ,\quad t = 0,1, \ldots ,K - 1$$

Here K is the IFFT size and j is the imaginary unit. The simulator sets the per-symbol bias to *s*_DC_ =  − min_*n*_ x[n].

8 $$s_{{{\mathrm{DC}}}} = - \mathop {{\mathrm{min}}}\limits_{t} s\left( t \right),\quad s_{b} \left( t \right) = s\left( t \right) + s_{{{\mathrm{DC}}}} ,\quad s_{{{\mathrm{clip}}}} \left( t \right) = {\mathrm{max}}\left( {0,s_{b} \left( t \right)} \right)$$

The biased waveform is non-negative, so the subsequent zero-clipping operation is inactive and the residual clipping-noise term is zero in the reported runs. A cached waveform-derived bias-normalization factor is applied to the beam power. Finite LED headroom, nonlinear transfer characteristics, quantization, and a fixed-bias clipping trade-off are not modeled.

The received signal *r*_*k*_(*t*) on subcarrier *k* contains the desired term, interference from other base stations, and additive noise:

9 $$r_{k} \left( t \right) = \eta \left( {G_{{0,{\mathrm{k}}}} s_{{0,{\mathrm{k}}}} \left( t \right) + \mathop \sum \limits_{i \in I} G_{{{\mathrm{i}},{\mathrm{k}}}} s_{{{\mathrm{i}},{\mathrm{k}}}} \left( t \right)} \right) + z_{k} \left( t \right)$$

Here, s_0,k_(t) is the transmitted signal sample from the serving base station on subcarrier k at time t, G0 and Gi are the channel gains from the serving and interfering base stations, η is the photodiode responsivity, I is the set of interfering base stations, and *zk*(t) is additive white Gaussian noise on subcarrier k.

### Macrocell-level statistical reporting

Every user is served by one beam-defined region; no coherent or non-coherent combining of the seven ADT beams is assumed. Let *S*_*u*_ denote the tones assigned to user u and *U*_*m*_ the active users evaluated in macrocell m. The per-user mean linear SINR is

10 $$\gamma_{u} = \frac{1}{{\left| {{\mathcal{S}}_{u} } \right|}}\mathop \sum \limits_{{s \in {\mathcal{S}}_{u} }} \gamma_{u,s}$$

The macrocell mean reported in decibels is computed after averaging the linear user values:

11 $${\mathrm{SINR}}_{{{\mathrm{mean}},m}} \left[ {{\mathrm{dB}}} \right] = 10{\mathrm{log}}_{10} \left( {\frac{1}{{\left| {{\mathcal{U}}_{m} } \right|}}\mathop \sum \limits_{{u \in {\mathcal{U}}_{m} }} \gamma_{u} } \right)$$

With *Ω*_*u*_ = *Σs**∈**S*_*u*_ log_2_(1 + *γ*_*u,s*_), the mean aggregate user spectral efficiency is

12 $$\overline{\Omega}_{m} = \frac{1}{{\left| {{\mathcal{U}}_{m} } \right|}}\mathop \sum \limits_{{u \in {\mathcal{U}}_{m} }} \Omega_{u}$$

Fairness is summarized from the same aggregate user values as

13 $$J_{m} = \frac{{\left( {\mathop \sum \nolimits_{{u \in {\mathcal{U}}_{m} }} \Omega_{u} } \right)^{2} }}{{\left| {{\mathcal{U}}_{m} } \right|\mathop \sum \nolimits_{{u \in {\mathcal{U}}_{m} }} \Omega_{u}^{2} }}$$

Thus, the macrocell quantities are arithmetic summaries of physical serving-beam links from Eqs. (5)–(6), not channel-gain or power combinations across beams.

The same rule is used for all static tables and figures: serving-link values are formed first and only then pooled for statistical reporting.

## Evaluation metrics

This section defines serving-link and statistical-summary metrics. Static rr1 values describe the active links sampled in independent trials; temporal-user service metrics are reserved for the dynamic experiment.

For each allocated tone, *γ*_*u,s*_ = *S*_*u,s*_/(*I*_*u,s*_ + *N*_*s*_), because the adopted dynamic bias gives *N*_*clip,u,s*_ = 0 in the reported static runs. Equations (10)–(13) summarize these serving-link values across tones and users.

14 $${\mathrm{SINR}}_{s} = \frac{{S_{s} }}{{I_{s} + N_{s} }}$$

Here *S*_*u,s*_, *I*_*u,s*_, and *N*_*s*_ are the desired electrical signal power, accumulated co-channel interference, and per-subcarrier thermal-noise variance.

The Shannon expression is used here as a comparative spectral-efficiency mapping from SINR to bps/Hz^39^. For IM/DD optical wireless links, the non-negativity of the transmitted waveform, peak-power constraints, clipping, and LED/receiver non-idealities mean that this expression should be interpreted as an upper-bound-style benchmark rather than an exact hardware capacity formula. This convention is sufficient for comparing the protocol variants under identical assumptions, but the absolute SE values should not be interpreted as experimentally achievable rates without implementation-specific modulation, coding, and device constraints.

15 $${\mathrm{SE}}_{s} = {\mathrm{log}}_{2} \left( {1 + {\mathrm{SINR}}_{s} } \right)$$

The aggregate user quantity *Ω*_*u*_ is a sum of per-tone spectral efficiencies over *S*_*u*_. It is not a conventional bandwidth-normalized link rate. A rate would require the subcarrier spacing and scheduling airtime, for example *R*_*u*_ = Δf *Σs**∈**S*_*u*_ log_2_(1 + *γ*_*u,s*_).

16 $${\mathrm{SE}}_{{{\mathrm{user}}}} = \mathop \sum \limits_{{s \in S_{u} }} {\mathrm{log}}_{2} \left( {1 + {\mathrm{SINR}}_{s} } \right)$$

Coverage at threshold γ is the fraction of sampled active links whose mean assigned-tone SINR exceeds γ. Jain’s index is computed over the same sampled *Ω*_*u*_ values^40^. In the static rr1 regime, it is a sampled-link dispersion index, not temporal fairness among persistent users.

## Experiments and Monte Carlo methodology

This section records the source-truth implementation choices needed to reproduce and correctly interpret the archived outputs.

The archived static pipeline exports full_protocol_summary.csv, sensitivity_delta.csv, sensitivity_users.csv, sensitivity_rx_tilt.csv, and comparison_prior_schemes.csv. The static scripts do not persist fixed seeds or raw independent trial summaries, so the archived tables are point estimates rather than paired statistical tests.

The custom Python pipeline contains topology generation, LOS gain, user dropping, static resource allocation, edge-pool coloring, sensitivity drivers, and CSV export. The DOI archives and exact release identifiers are given in Data and Code Availability.

Algorithm 1 summarizes the proposed subcarrier allocation procedure.

**Algorithm 1:**
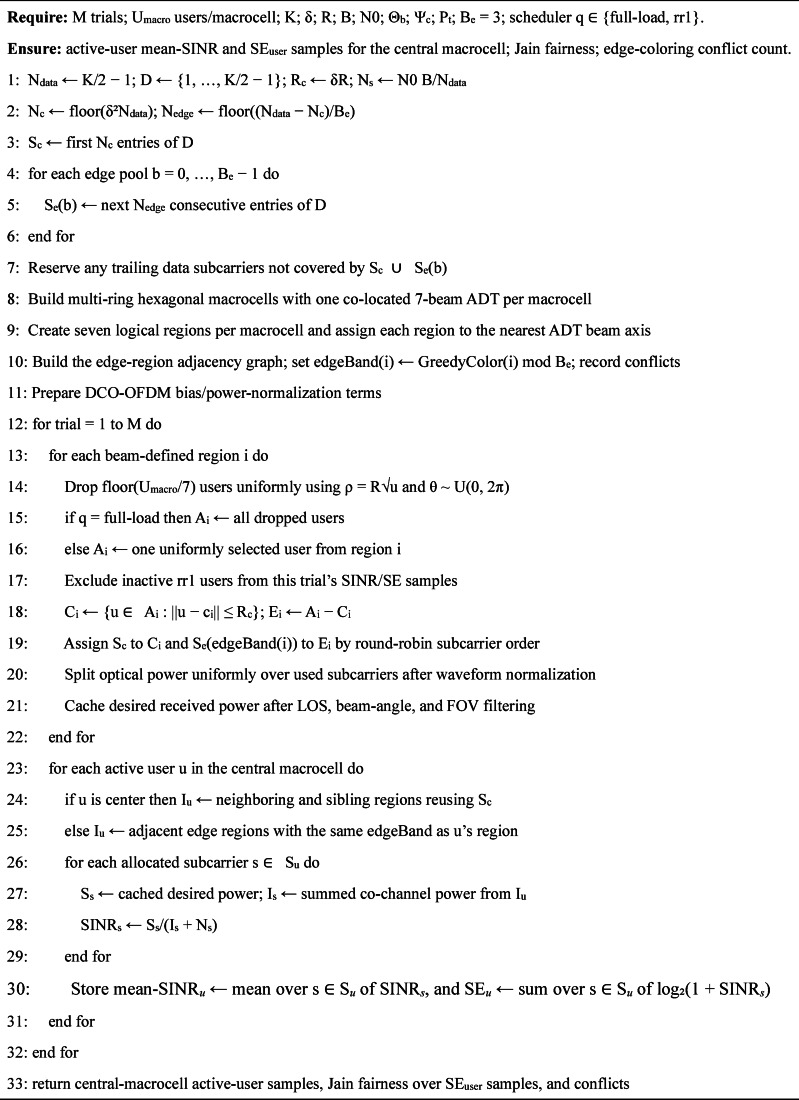
Proposed ADT-OFDMA-strict-FFR simulation kernel

**Algorithm 2:**
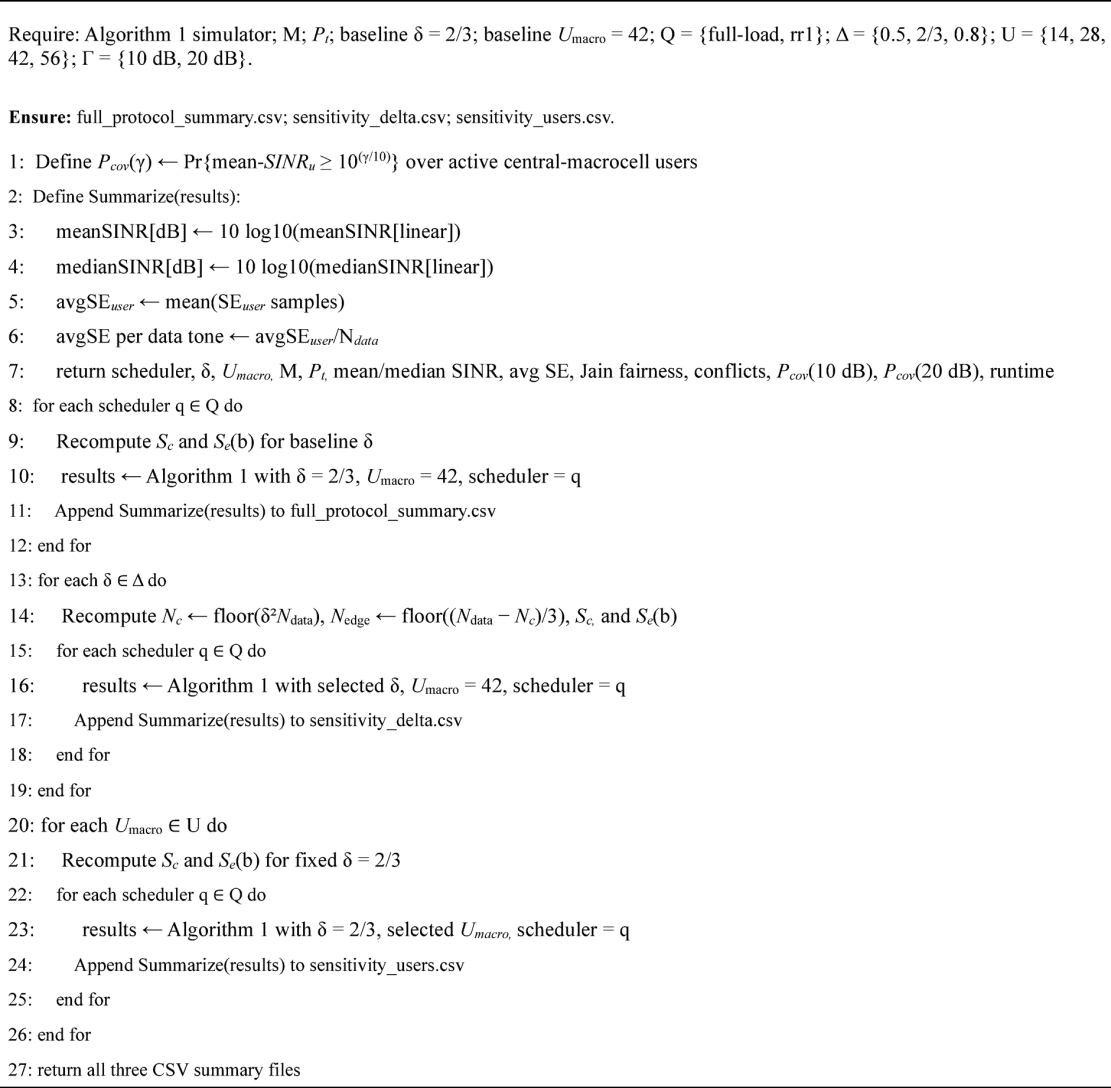
Full-protocol experiment driver

For the static implementation, the dominant loop cost is O(M·U·*N*_sc_·*N*_int_). In the canonical protected baseline, an auxiliary source-instrumented audit found no positive accumulated co-channel term in 3,902 full-load and 1,130 rr1 evaluated tone samples over 500 seeded trials per regime; the archived joint result therefore does not substantiate a uniformly interference-limited label. Power sensitivity and configuration-specific interpretation are retained.

### Implementation and coordination scope

The reported complexity characterizes the offline simulation pipeline and the protocol-level resource assignment evaluated in this study. The static benchmark assumes centralized knowledge of user positions, serving-region membership, and edge-region adjacency at the beginning of each Monte Carlo trial. The dynamic extension in “Dynamic reliability-aware extension under mobility and NLOS stress” Section similarly assumes centralized frame-level access to simulated user state, channel-state indicators, service-gap counters, transition-risk terms, and the information required for variable multi-user admission, OFDMA sharing, and normalized regional power allocation. These assumptions are appropriate for evaluating the scheduling and allocation rules under controlled conditions, but they do not prescribe a distributed AP-level implementation with limited local observations and inter-AP message exchange.

### Average spectral efficiency and simulation setup

#### Theoretical spectral efficiency

Per-tone log_2_(1 + γ) is used only as a common comparative mapping. The user aggregate is the sum over allocated tones; it should not be described as long-term throughput without subcarrier spacing and airtime.

17 $$C = {\mathrm{log}}_{2} \left( {1 + {\mathrm{SINR}}} \right)$$

Each user’s SINR is produced by the declared Lambertian LOS channel, FOV/beam gates, thermal noise, and the co-channel terms that remain under the selected resource variant.

18 $$\Omega = \mathop \smallint \limits_{0}^{\infty } {\mathrm{log}}_{2} \left( {1 + \gamma } \right)f_{\Gamma } \left( \gamma \right)\,d\gamma$$

Monte Carlo sampling approximates the resulting empirical distributions; no closed-form SINR density is claimed.

The static experiment uses 10,000 independent spatial trials for each canonical configuration.

19 $$\Omega \approx \frac{1}{M}\mathop \sum \limits_{i = 1}^{M} {\mathrm{log}}_{2} \left( {1 + {\mathrm{SINR}}_{i} } \right)$$

The resulting means, medians, coverage fractions, and sampled-link indices are point estimates for the stated model.

#### Simulation setup and assumptions

Two static loading regimes are considered: full-load activates all dropped users, whereas rr1 selects one user uniformly in each region in each independent trial. rr1 is not a persistent-user round-robin process.

Table 1 lists the source-truth baseline parameters. The static topology uses seven co-located beam axes spaced by 360°/7 = 51.43°, a 20° transmitter-side selection gate, and a 40° receiver FOV. The dynamic extension uses the distinct central-plus-six-outer geometry and parameters listed in Table 6.

Those beam-defined regions are used for user dropping, beam selection, and adjacency-aware reuse assignment.

The simulator uses K = 512 subcarriers per microcell, so Ndata = K/2 − 1. The center pool size is Nc = ⌊δ^2^ Ndata⌋, and the remaining subcarriers are divided among three protected edge pools following the strict-FFR resource partitioning described in ^6,7^. The protected edge pools are assigned to the edge regions using the adjacency-aware greedy graph-coloring rule defined in “Edge-pool graph coloring implementation” Section. The edge-pool graph-coloring conflict count is recorded in the simulation output; it is zero for the baseline and reported sensitivity configurations.

Hermitian-symmetric QPSK DCO-OFDM is used in the static waveform-normalization routine.

Users are dropped uniformly within the beam-defined regions, assigned to the best-aligned beam, classified as center or edge according to *R*_*c*_ = δ*R*, and filtered by the receiver FOV *Ψ*_*c*_.

For each distinct active-tone count, the simulator creates one QPSK/Hermitian waveform, computes x[n], and sets *s*_DC_ = −min_*n*_ x[n]. Since the biased samples are non-negative, zero clipping is inactive and residual clipping noise is zero.

Center users reuse the common center pool in all cells, while edge users use protected pools that are not reused by adjacent edge regions.

### Source-truth consistency and power/interference audit

The archived full_protocol_summary.csv is the canonical static reference: mean SINR is 28.11 dB under full load and 28.28 dB under rr1, with medians 27.10 and 27.15 dB. These values are reproducibility targets for the implementation, not an independent analytical validation. A 500-trial source-instrumented audit (seed 20,260,712) observed zero accumulated co-channel interference in every evaluated joint-baseline tone sample; therefore the protected baseline is not described as demonstrably interference-limited.

### Monte Carlo uncertainty and accurate reporting

The static archive preserves aggregate CSV summaries but not raw independent seed batches or trial-level cluster summaries. Static tables are therefore reported as point estimates. A binomial Wald interval using 10,000 as though every pooled user sample were independent would be unjustified, and it degenerates when ${\hat{\mathrm{p}}}$ = 1.000. No static confidence interval or significance test is claimed.

For the dynamic experiment, the independent unit is the random seed. Table 7 reports the mean across ten seed summaries with 95% confidence half-width 1.96 s/√10. Values of 1.000 in the static tables mean only that no failure was observed in the finite archived run.

## Results and discussion

### SINR behavior and interference characteristics

**Fig. 4:**
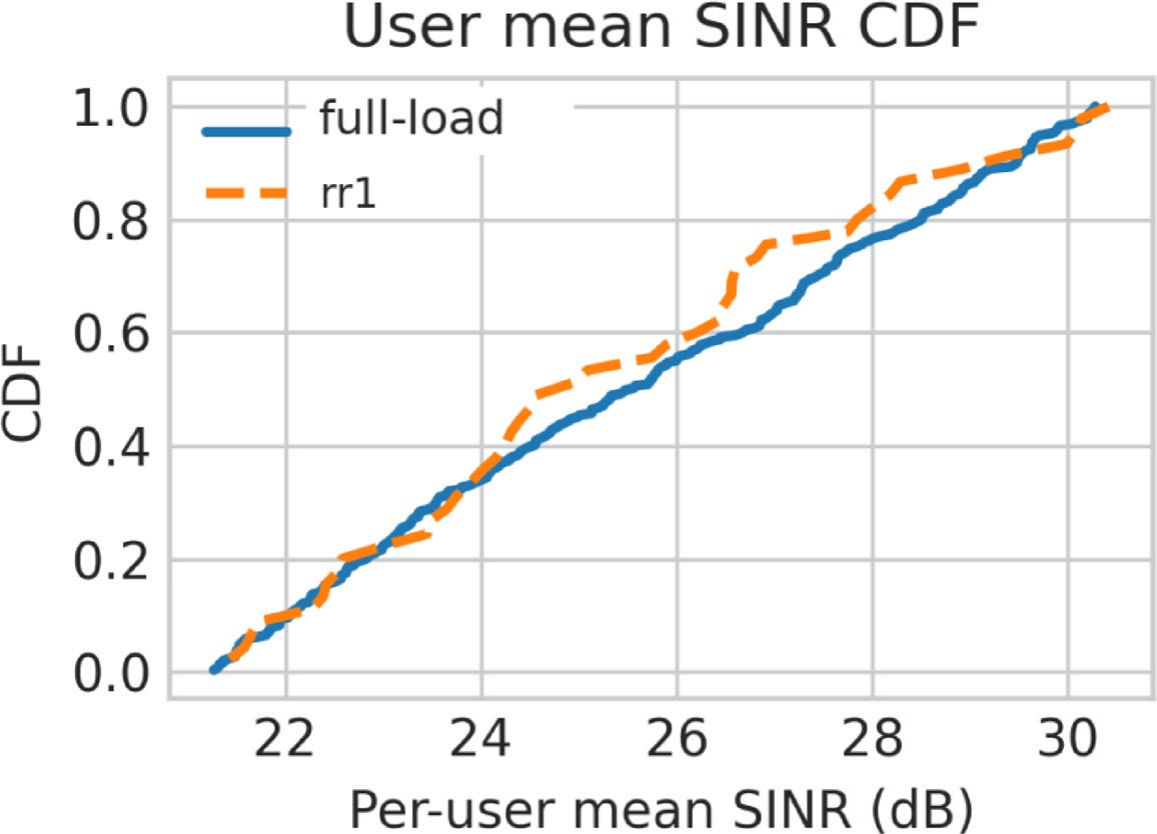
Per-user mean-SINR CDF under full-load and rr1 scheduling.

**Fig. 5:**
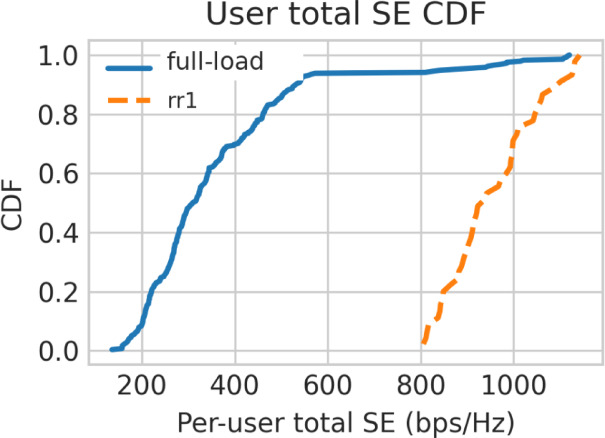
Per-user aggregate spectral-efficiency CDF under full-load and rr1 scheduling.

**Fig. 6:**
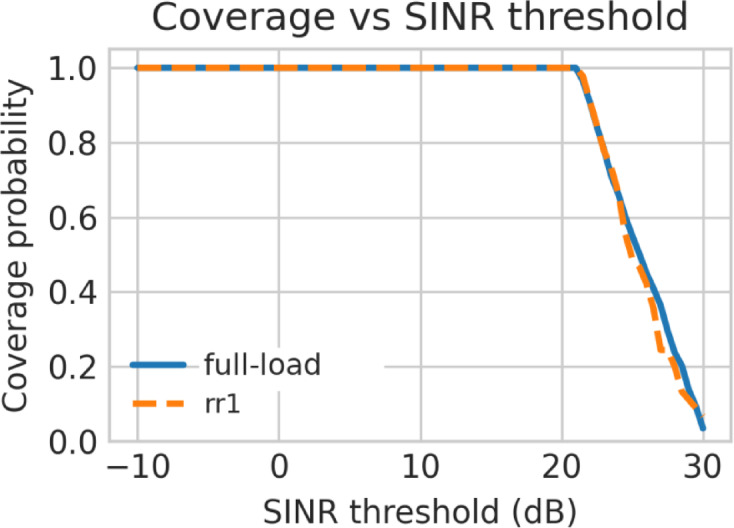
Coverage probability versus SINR threshold under full-load and rr1 scheduling.

**Fig. 7:**
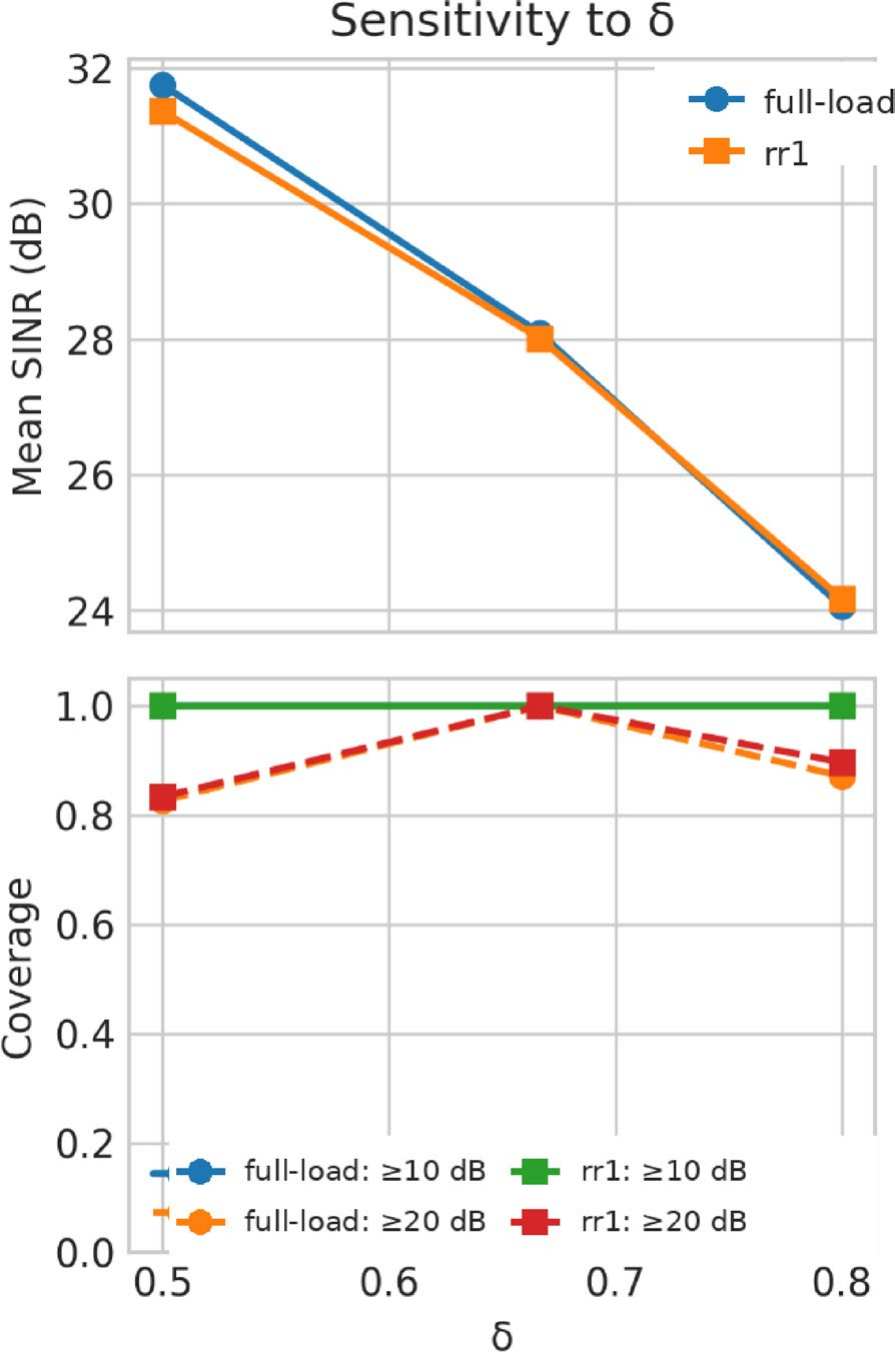
Sensitivity to δ.

**Fig. 8:**
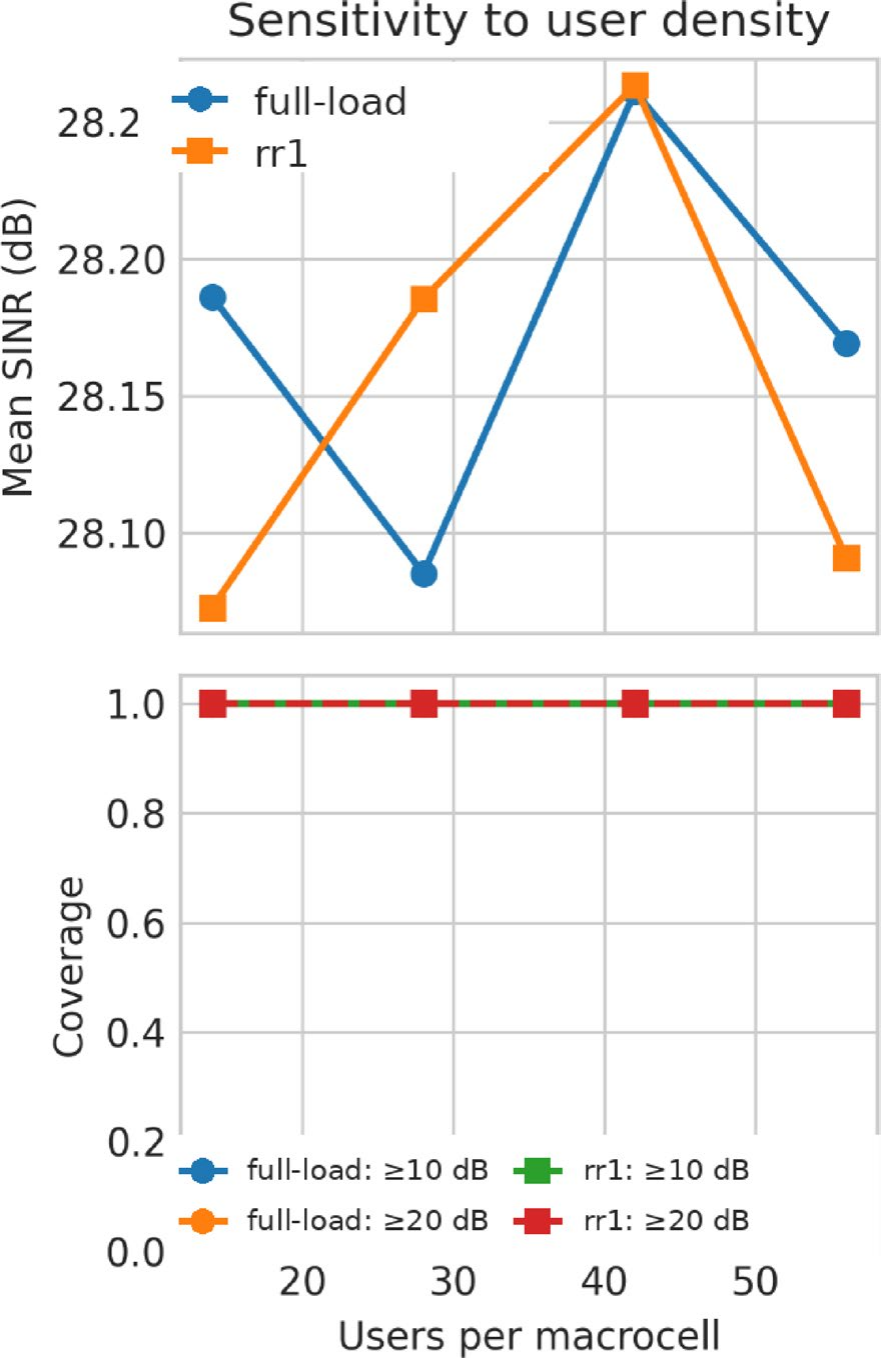
User-density sensitivity.

Figures 4, 5 and 6 compare the two static active-link sampling regimes; Figs. 7 and 8 report parameter sensitivity. The spread in Fig. 4 reflects a mixture of positions, angular alignments, and center/edge classes; the two scheduler-level curves do not by themselves identify separate center and edge distributions.

The edge-sub-band graph-coloring conflict count was zero in the baseline and all reported center-partition and user-density sensitivity configurations, confirming that adjacent edge regions were not assigned the same protected edge pool in the evaluated hexagonal layout.

This trend is consistent with the modeling assumptions used in the simulator. Desired links benefit from stronger beam alignment, while the strongest edge interferers are removed by the protected-edge assignment. The remaining spread in Fig. 4 is therefore mainly explained by user position within the beam-defined region, angular mismatch, and the difference between center and edge operation^17,18^.

The median SINR remains noticeably lower than the upper tail, which is expected in a beam-defined multi-cell layout. Center users usually see shorter effective paths and better alignment with the serving beam, whereas edge users operate closer to the region boundary and are more exposed to residual co-channel terms. For that reason, the SINR distribution should not be read as uniformly high for all users, even though the protocol suppresses the dominant adjacent-edge interference terms.

### Spectral efficiency and throughput trends

Figure 5 reports *Ω*_*u*_ = Σ log_2_(1 + γ) over the tones allocated to each sampled active link. rr1 users receive a larger tone share in the selected trial, so the conditional active-link distribution shifts upward.

This difference is an instantaneous resource-sharing effect. It is not evidence of nearly threefold long-term user throughput because user identities, queues, duty cycle, subcarrier spacing, and repeated scheduling airtime are absent from the static experiment.

The adopted per-symbol bias prevents clipping in the reported runs, so the curves cannot be used to infer clipping robustness. Hardware nonlinearities and a fixed-bias clipping trade-off require a separate model.

### Coverage probability across SINR thresholds

Figure 6 shows the coverage probability obtained from per-user mean SINR. A large share of users remains above moderate thresholds, and the drop at stricter thresholds is gradual rather than abrupt. This is consistent with the geometry of the model: the protocol protects the strongest edge-interference paths, but users near beam boundaries still experience weaker alignment and therefore lower SINR margins.

Coverage is controlled by the lower tail of the SINR distribution. In this model, that tail is improved by two specific mechanisms: adjacent edge regions do not reuse the same protected pool, and receiver FOV filtering removes part of the off-axis optical energy that would otherwise contribute to interference.

The very strong values at 20 dB should therefore be interpreted carefully. In this manuscript, coverage is computed from per-user mean SINR, not from worst-subcarrier outage or packet-level reliability. The reported values reflect a favorable protocol-level regime built on LOS-only propagation, beam-selective ADT transmission, strict FFR, receiver FOV filtering, and simplified scheduling assumptions. They show what the protocol can achieve under that controlled model, not a universal guarantee for arbitrary LiFi deployments with mobility, NLOS propagation, or hardware non-idealities.

### Fairness behavior

Static Jain indices are computed over pooled active-link *Ω*_*u*_ samples. In rr1, one uniformly random user is selected in each region and users are redropped in the next trial; J = 0.990 is therefore sampled-link dispersion, not temporal fairness among persistent users.

The full-load value J = 0.793 is defined over simultaneous active-link samples and partly reflects the fixed equal-resource allocation. It is not the maximum fairness achievable by an adaptive scheduler.

Persistent-user service gaps and time-varying fairness are evaluated only in the dynamic experiment, where unscheduled users receive zero frame rate.

Accordingly, the static comparison separates active-link loading and resource-share distributions; it does not establish long-term scheduler fairness.

### Sensitivity study (scheduler regimes)

Figures 7 and 8 summarize the two sensitivity sweeps. We vary the center/edge partition ratio δ *∈* {0.5, 2/3, 0.8} and the offered load by changing the number of users per macrocell (14, 28, 42, 56), and we report both full-load and rr1 so that scheduler effects remain visible.

Changing δ alters both the classification radius and the center-pool size *N*_*c*_ = ⌊δ^2^ *N*_*data*_⌋, so it directly changes how much spectrum is placed under full reuse versus protected reuse. A larger center region increases reuse efficiency but also exposes more users to center-pool interference; a smaller center region protects more edge users but reduces the aggressively reused portion of the spectrum.

The δ sweep is included to make the center/edge trade-off explicit and to avoid implying that a single partition ratio is optimal for all room sizes, receiver FOVs, powers, or user densities. In the considered configuration, δ = 2/3 provides a balanced operating point, but a deployment-specific optimization of δ is outside the scope of this static benchmark.

Figure 7 shows this trade-off clearly. At δ = 0.5, the mean SINR is 31.75 dB (full-load) and 31.37 dB (rr1), whereas at δ = 0.8 it falls to 24.06 dB (full-load) and 24.17 dB (rr1). The intermediate setting δ = 2/3 gives the most balanced outcome in the present setup, with strong SINR margin and no observed 20 dB outage in the finite LOS-static sample under both regimes.

Increasing offered users from 14 to 56 leaves the sampled active-link SINR near 28 dB. Under rr1, the selected link continues to obtain the regional pool, so conditional aggregate SE and the sampled-link Jain index remain high. Under full load, more simultaneous users divide the pool. These trends must not be read as long-term rr1 throughput or fairness (Table 2).

**Table 2:** Canonical archived static summary (central-macrocell active links, δ = 2/3, 42 users per macrocell, 10,000 trials). rr1 is random-one-per-region sampling with redropped users; its aggregate SE and Jain index are conditional sampled-link metrics, not persistent-user throughput or temporal fairness. Coverage 1.000 means no failure observed in the finite run. Static confidence intervals are not available from the archived aggregate CSV summaries.

Metric	Full-load	rr1
Mean SINR (dB)	28.11	28.28
Median SINR (dB)	27.10	27.15
P(mean SINR ≥ 10 dB)	1.000	1.000
P(mean SINR ≥ 20 dB)	1.000	1.000
Aggregate SE/*N*_data_	1.474	4.035
Aggregate per-active-link SE, Σ log_2_(1 + γ)	375.75	1028.88
Jain index over active-link samples	0.793	0.990
Adjacent same-edge-pool conflict count	0	0

### Orientation sensitivity

The baseline configuration in “Assumptions” Section assumes an upward-facing receiver with the photodiode normal aligned to the ceiling, so every user that lies inside a beam cone is served under the ideal incidence angle. This is a best-case assumption that is known to inflate coverage in LiFi studies once random receiver orientation and handheld-device tilt are considered^41^.

Table 3 shows strong orientation sensitivity. At σ = 29°, mean SINR falls to 18.46 dB under full load and 19.12 dB under rr1, while 20 dB coverage falls below 0.11. rr1 retains larger conditional aggregate SE, but its sampled-link Jain index is not a temporal-user fairness measure.

**Table 3:** Orientation sensitivity at δ = 2/3, 42 users per macrocell, *P*_*t*_ = 3 W, and 10,000 trials. σ is the polar-tilt standard deviation. Values are archived point estimates; no static confidence intervals are claimed.

σ (deg)	Scheduler	Mean SINR (dB)	Median SINR (dB)	P(SINR ≥ 10 dB)	P(SINR ≥ 20 dB)	Jain index	Aggregate per-active-link SE, Σ log_2_(1 + γ)
5	Full-load	27.32	26.39	1.000	0.975	0.786	357.68
5	rr1	27.38	26.32	1.000	0.979	0.977	980.70
15	Full-load	24.23	20.73	0.715	0.364	0.469	170.44
15	rr1	24.30	16.74	0.745	0.365	0.622	498.55
29	Full-load	18.46	5.10	0.348	0.094	0.330	80.38
29	rr1	19.12	6.15	0.366	0.101	0.445	248.90

## Comparison with related work

Table 4 differentiates representative mechanism families, while Table 5 reports controlled internal variants. The variants are not reproductions of^5–13^ and do not support cross-paper superiority claims.

**Table 4:** Protocol-level comparison between the proposed scheme and representative literature.

Aspect	Proposed scheme vs representative literature
Multiple access	This work: ADT-enabled SDMA + OFDMA with strict FFR. Prior: ADT-SDMA in optical attocells^5^; OFDMA VLC baselines^8–10^
Reuse/FFR	This work: strict FFR (common center band + protected edge bands) with conflict-free assignment. Prior: sFFR/SFR in DCO-OFDM optical attocells^6,7^
Impairments	Dynamic per-symbol DC bias; no residual clipping in reported runs; finite LED headroom and LED nonlinearity excluded
Noise model	PSD-based thermal-noise variance per data tone; co-channel terms accumulated as implemented; no clipping-noise term in reported runs
Evaluation regime	This work reports both full activity ('full-load’) and scheduled (‘rr1’) regimes to expose interference-loading sensitivity, consistent with scheduler-driven evaluations^10^

**Table 5:** Controlled static variants at 42 users per macrocell and *P*_*t*_ = 3 W. Joint rows reproduce the canonical Table 2 baseline. ‘No FFR’ uses δ = 1. 'Angular selectivity relaxed’ jointly widens the transmitter gate and receiver FOV. Variant estimates are unpaired and are not exact reproductions of prior studies.

Scheme	Scheduler	Mean SINR (dB)	Median SINR (dB)	P(SINR ≥ 10 dB)	P(SINR ≥ 20 dB)	Aggregate per-active-link SE, Σ log_2_(1 + γ)	Jain fairness
Joint (canonical)	Full-load	28.11	27.10	1.000	1.000	375.75	0.793
Joint (canonical)	rr1	28.28	27.15	1.000	1.000	1028.88	0.990
No FFR (δ = 1)	Full-load	20.24	19.22	1.000	0.416	274.87	0.982
No FFR (δ = 1)	rr1	20.06	19.05	1.000	0.404	1638.87	0.983
FFR retained; angular selectivity relaxed	Full-load	3.78	− 19.96	0.031	0.003	9.90	0.052
FFR retained; angular selectivity relaxed	rr1	4.56	− 16.17	0.032	0.006	32.52	0.080
No FFR; angular selectivity relaxed	Full-load	− 3.88	− 23.65	0.012	0.000	7.79	0.083
No FFR; angular selectivity relaxed	rr1	− 3.95	− 23.55	0.011	0.000	46.45	0.084

Relative to the canonical joint baseline, disabling strict FFR by setting δ = 1 lowers mean SINR by 7.87 dB under full load and 8.22 dB under rr1; rr1 20 dB coverage falls by 59.6 percentage points. The no-FFR rr1 variant has larger conditional aggregate SE because the selected link accesses the unpartitioned pool, but it loses protected-edge coverage. Relaxing transmitter and receiver angular filtering produces much larger losses.

Ablation scope. The no-FFR row is a compound δ = 1 variant that changes classification and resource partition. The 'angular selectivity relaxed’ rows jointly widen the transmitter gate and receiver FOV, so they do not isolate a transmitter-only ADT effect. The joint rows reuse Table 2's canonical archived estimates; the remaining rows are separate, unpaired Monte Carlo point estimates.

## Dynamic reliability-aware extension under mobility and NLOS stress

The preceding sections establish the static ADT-SDMA-OFDMA strict-FFR benchmark. To address concerns regarding mobility, NLOS effects, handover-related service disruption, and practical multi-user scheduling, this section adds a dynamic frame-level layer while preserving the beam-defined microcells, the fixed center/edge partition δ = 2/3, and the strict-FFR edge-pool assignment. The extension recomputes user and channel state at every frame, ranks candidate users, and admits a variable number of users to orthogonal subcarriers within each regional pool. This keeps the static framework as the reproducible baseline while giving OFDMA an explicit intra-region multiple-access role under mobility, orientation variation, first-order NLOS stress, and service-continuity constraints.

**Fig. 9:**
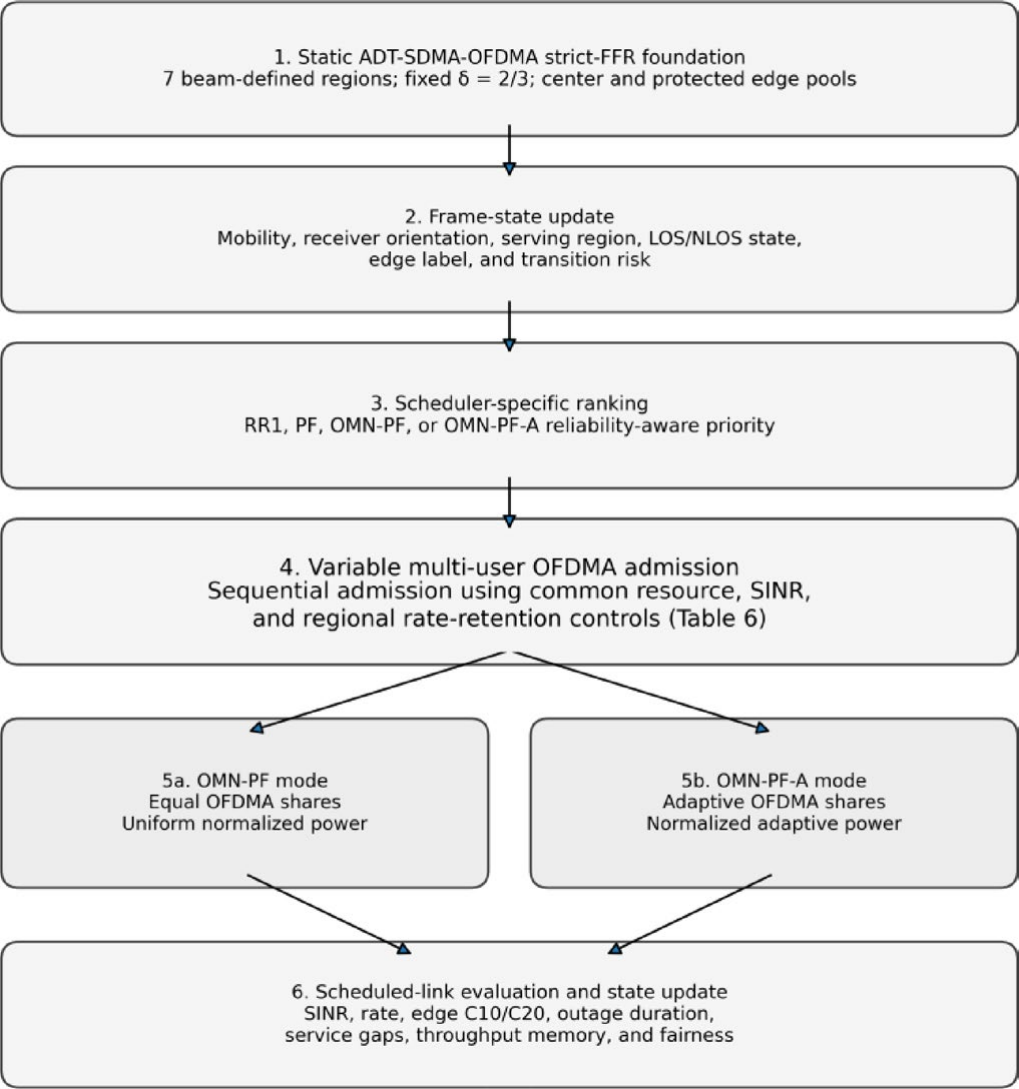
Dynamic extension workflow. RR1 and PF retain their baseline rankings; only OMN-PF and OMN-PF-A use the reliability-aware priority. All four policies use the common admission test.

The main comparison considers RR1, conventional PF, OMN-PF, and OMN-PF-A under the same variable multi-user admission constraints. OMN-PF uses the reliability-aware ranking metric with equal OFDMA sharing and uniform normalized regional power. OMN-PF-A uses the same ranking and admission framework but applies bounded adaptive OFDMA sharing and normalized adaptive power redistribution. The complete dynamic processing workflow is summarized in Fig. 9. The purpose is not to claim universal optimality, but to identify two operating modes: a balanced lower-complexity OMN-PF mode and an adaptive mode that emphasizes high-threshold edge continuity. This framing is consistent with prior LiFi networking and mobility studies, which identify user movement, beam switching, resource allocation, and coordination overhead as major challenges in dense indoor optical networks^10,42–45^.

Notation used in “Dynamic reliability-aware extension under mobility and NLOS stress” Section. Unless stated otherwise, *u* indexes users, *t* indexes frames, *b* indexes beam-defined regions, *p* indexes a center or edge resource pool, and *q* indexes reflecting patches. A hat denotes a pre-admission prediction, a bar denotes a moving average, and *1{·}* is the indicator function.

### Dynamic frame model and user mobility

**Fig. 10:**
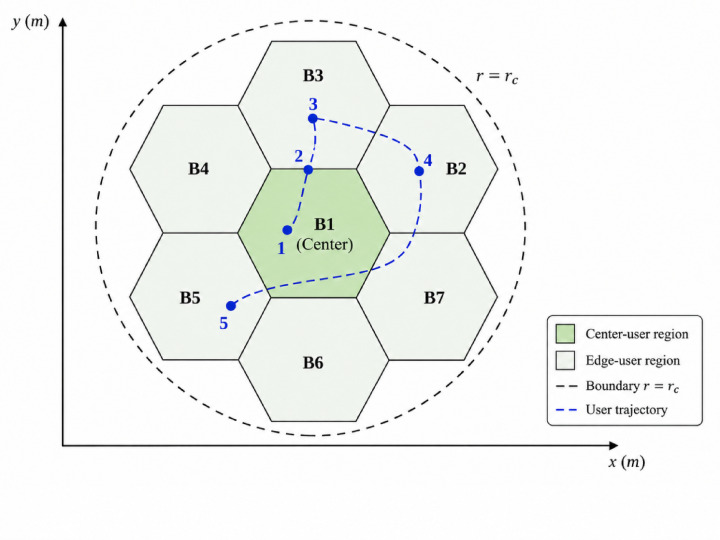
Conceptual seven-region beam-defined layout and representative mobility trajectory. The center region and six surrounding regions illustrate center/edge transitions and serving-region changes; the numerical dynamic geometry and association parameters are specified in “Dynamic frame model and user mobility” Section and Table 6.

At frame *t*, user *u* is described by the horizontal position *p*_*u*_(t) = [*x*_*u*_(t), *y*_*u*_(t)]ᵀ, receiver normal *n*_*u*_(t), serving region *s*_*u*_(t), center/edge indicator *E*_*u*_(t), moving-average rate $\overline{R}_{u} \left( {\mathrm{t}} \right)$, and service-gap counter *G*_*u*_(t). The frame duration is Δt = 0.1 s, and the walking speed *v*_*u*_(t) is sampled from U(0.2, 1.5) m/s inside the 16 × 16 m room.

20 $$x_{u} \left( {t + \Delta t} \right) = x_{u} \left( t \right) + v_{u} \left( t \right){\mathrm{cos}}\omega_{u} \left( t \right)\Delta t,$$

21 $$y_{u} \left( {t + \Delta t} \right) = y_{u} \left( t \right) + v_{u} \left( t \right){\mathrm{sin}}\omega_{u} \left( t \right)\Delta t.$$

where ${\omega}_{u}(t)$ denotes the user’s direction of movement in the horizontal plane. A new waypoint and speed are selected when a waypoint is reached, and boundary handling keeps the trajectory inside the room. The process is a controlled mobility stress model that creates serving-region and center/edge transitions; it does not model lower-layer handover signaling, buffering, or packet transfer (Fig. 10).

**Table 6:** Source-truth settings for the dynamic v1.1.1 experiment.

Parameter group	Meaning	Value used
Run	Independent experiment structure	10 seeds × 300 trials × 75 frames; 42 users; Δt = 0.1 s
Schedulers	Policies reported	RR1, PF, OMN-PF, OMN-PF-A
Resources	Fixed strict-FFR numerology	δ = 2/3; K/N_data_/N_c_/N_e_ = 512/255/113/47; static edge coloring
Dynamic beams	FOV and beam profiles	Ψ_c_ = 50°; outer angular σ = 24°; central spatial σ = 3 m
Room/mobility	Random waypoint	16 × 16 m; TX/RX heights = 3 m/0.85 m; speed 0.2–1.5 m/s
Orientation	Initial and correlated updates	Initial 15° ± 4°; ρ = 0.85; tilt innovation 5°; azimuth 10°; clip 0–80°
Reflection stress	Patches and scaling	8 wall + 4 floor; ρ_wall_ = 0.72; ρ_floor_ = 0.32; NLOS × 3; LOS decay 28°; edge factor 0.65
Memory/ranking	PF and reliability weights	η = 0.10; G_max_ = 10; α = 0.20; λ_o/e/b/n/s_ = 2/14/0.5/8/6
Outage state	Logistic and memory	target 10 dB; scale 2.5 dB; memory 0.8/0.2; boundary scale 0.30R
Admission	Common feasibility controls	Nmin = 16; γ_adm_ = 10 dB; β_R_ = 0.55; ceilings 7 center/2 edge/9 total
OMN-PF-A	Bounded adaptation	OFDMA [0.75, 4.0]; channel [0.75, 1.50]; reliability ≤ 1.75; normalized regional power

### Receiver-orientation update

The receiver orientation is represented by the unit normal vector *n*_*u*_(t) in Eq. (22). Here *θ*_*u*_(t) is the polar tilt measured from the upward vertical axis, and *φ*_*u*_(t) is the receiver azimuth in the horizontal plane. This vector is used to calculate the incidence angle and determine whether the optical path lies inside the receiver FOV.

22 $${\mathbf{n}}_{u} \left( t \right) = \left[ {{\mathrm{sin}}\theta_{u} \left( t \right){\mathrm{cos}}\phi_{u} \left( t \right),\,{\mathrm{sin}}\theta_{u} \left( t \right){\mathrm{sin}}\phi_{u} \left( t \right),\,{\mathrm{cos}}\theta_{u} \left( t \right)} \right].$$

Equation (23) updates the tilt through a correlated mean-reverting process. The coefficient *ρ* = 0.85 controls temporal correlation, *μ*_*θ*_ = 15° is the mean tilt, and *ε*_*t*_ is a zero-mean Gaussian tilt innovation with 5° standard deviation. The result is clipped to [0°, 80°]. Equation (24) updates the azimuth, where *ν*_*t*_ is a zero-mean Gaussian azimuth innovation with 10° standard deviation and the angle is wrapped modulo 360°. The initial tilt is drawn from a clipped N(15°, (4°)2) distribution.

23 $$\theta_{u} \left( t \right) = \rho \theta_{u} \left( {t - 1} \right) + \left( {1 - \rho } \right)\mu_{\theta } + \epsilon_{t} .$$

24 $$\phi_{u} \left( t \right) = \phi_{u} \left( {t - 1} \right) + \nu_{t} .$$

The correlation coefficient prevents unrealistically independent orientation jumps between adjacent frames. The resulting orientation state changes the receiver incidence angle, FOV visibility, LOS gain, and the orientation attenuation applied in the NLOS-stress profile. It is a parametric stress process rather than a fitted device-orientation trace.

### First-order NLOS stress model

The dynamic experiment uses a parametric first-order reflected-path stress model rather than calibrated ray tracing or a measurement-derived NLOS channel. Equation (25) gives the reflected channel contribution from transmitter beam *b* to user *u* through reflecting patch *q*.

25 $$H_{u,b,q}^{{{\mathrm{NLOS}}}} \left( t \right) = \frac{{\left( {m + 1} \right)A_{pd} \rho_{q} \Delta A_{q} }}{{2\pi^{2} d_{b,q}^{2} d_{q,u}^{2} \left( t \right)}}{\mathrm{cos}}^{m} \left( {\varphi_{b,q} } \right){\mathrm{cos}}\left( {\alpha_{q} } \right){\mathrm{cos}}\left( {\beta_{q} } \right)T_{s} g\left( {\psi_{q,u} \left( t \right)} \right){\mathrm{cos}}\left( {\psi_{q,u} \left( t \right)} \right)G_{b,q} .$$

In Eq. (25), *H*_*u,b,q*_^NLOS^(t) is the first-order reflected gain; *m* is the Lambertian order; *A*_*PD*_ is the photodiode area; *ρ*_*q*_ and Δ*A*_*q*_ are the reflection coefficient and area of patch *q*; *d*_*b,q*_ and *d*_*q,u*_(t) are the beam-to-patch and patch-to-user distances. The angles *φ*_*b,q*_, *α*_*q*_, and *β*_*q*_ describe emission toward, incidence on, and departure from the patch, while *ψ*_*q,u*_(t) is the incidence angle at the receiver. *T*_*s*_, *g*, and *G*_*b,q*_ denote optical-filter transmission, concentrator gain, and beam-pattern gain toward the patch, respectively. Eight wall patches and four floor patches are used.

26 $$H_{u}^{{{\mathrm{stress}}}} \left( t \right) = a_{u} \left( t \right)e^{{ - \theta_{u} \left( t \right)/28^{ \circ } }} H_{u}^{{{\mathrm{LOS}}}} \left( t \right) + 3\mathop \sum \limits_{q} H_{u,q}^{{{\mathrm{NLOS}}}} \left( t \right)$$

In Eq. (26), *H*_*u*_^stress^(t) is the effective dynamic channel used by the scheduler, *H*_*u*_^LOS^(t) is the direct LOS component, and Σ*H*_*u,q*_^NLOS^(t) is the sum of the first-order patch contributions. The factor *a*_*u*_(t) equals 0.65 for edge users and 1 otherwise; exp[− *θ*_*u*_(t)/28°] introduces orientation-dependent LOS attenuation; and the multiplier 3 deliberately strengthens the reflected component for stress testing. These factors are modeling controls, not measured room parameters.

### Beam-transition and handover-aware scheduling state

At every frame, the serving beam-defined region *s*_*u*_(t) is recomputed from the user position, beam geometry, and FOV state. Equation (27) defines *I*_*u*_^tr^(t) as a binary transition indicator: it equals one when the serving region differs from the previous frame and zero otherwise.

27 $${\mathrm{I}}_{{\mathrm{u}}}^{{{\mathrm{tr}}}} \left( {\mathrm{t}} \right) = {1}\left\{ {{\text{ s}}_{{\mathrm{u}}} \left( {\mathrm{t}} \right) \ne {\mathrm{s}}_{{\mathrm{u}}} \left( {{\mathrm{t}} - {1}} \right)} \right\}.$$

Let *d*_(1)_(t) and *d*_(2)_(t) denote the distances from user *u* to the nearest and second-nearest logical region centers. Their difference is small near a beam boundary and larger well inside a region. Equation (28) combines this geometric proximity with the actual transition indicator.

28 $$B_{u} \left( t \right) = {\mathrm{max}}\left\{ {{\mathrm{exp}}\left[ { - \frac{{d_{\left( 2 \right)} \left( t \right) - d_{\left( 1 \right)} \left( t \right)}}{0.30R}} \right],1\left[ {s_{u} \left( t \right) \ne s_{u} \left( {t - 1} \right)} \right]} \right\}$$

In Eq. (28), *B*_*u*_(t) is the beam-transition-risk score, *R* is the logical-region radius, and 0.30*R* is the boundary-proximity scale. The exponential term lies in (0, 1], and the maximum with 1{*s*_*u*_(t) ≠ *s*_*u*_(t − 1)} forces the risk to one in a frame where a region change occurs. This is a scheduling feature, not an explicit handover protocol or latency model.

### NLOS fragility, outage risk, and service gap

NLOS fragility is denoted *F*_*u*_^NLOS^(t) to avoid collision with the effective subcarrier count *n*_*u*_. Equation (29) defines it as the fraction of the combined LOS-plus-NLOS gain that is supplied by the first-order reflected component.

29 $$F_{u}^{{{\mathrm{NLOS}}}} \left( t \right) = \frac{{H_{u}^{{{\mathrm{NLOS}}}} \left( t \right)}}{{H_{u}^{{{\mathrm{LOS}}}} \left( t \right) + H_{u}^{{{\mathrm{NLOS}}}} \left( t \right) + \varepsilon }}$$

where ε is a small positive constant that avoids division by zero. Thus *F*_*u*_^NLOS^(t) approaches zero for LOS-dominant links and increases as the user becomes more dependent on reflected energy. The instantaneous outage risk *O*_*u*_(t) is a logistic function of the predicted SINR $\hat{y}_{u,dB} \left( {\mathrm{t}} \right)$ around the 10 dB target with a 2.5 dB transition scale. The memory state *O*_*mem,u*_(t) applies the 0.8/0.2 update to the observed link-outage indicator, and the scheduler uses the larger of the instantaneous and remembered risks.

30 $$S_{u} \left( t \right) = \frac{{{\mathrm{min}}\left\{ {G_{u} \left( t \right),10} \right\}}}{10}$$

Equation (30) defines the normalized service-gap term *S*_*u*_(t), where *G*_*u*_(t) is the number of consecutive frames for which user *u* has not been scheduled. The cap of 10 frames prevents the waiting-time contribution from increasing without bound; therefore *S*_*u*_(t) lies between zero and one.

### Service-gap-aware OMN-PF ranking metric

For each candidate user *u* in a beam-defined region, the scheduler first predicts the rate available from the strict-FFR OFDMA pool associated with the user’s current center/edge class. This is a pre-admission quantity; the actual SINR and served rate are recalculated after the admitted users share the pool.

31 $$\hat{R}_{u} \left( t \right) = \mathop \sum \limits_{{k \in {\mathcal{K}}_{u} \left( t \right)}} {\mathrm{log}}_{2} \left( {1 + \hat{\Gamma}_{u,k} \left( t \right)} \right).$$

In Eq. (31), $\hat{R}_{u} \left( {\mathrm{t}} \right)$ is the predicted aggregate rate, *K*_*u*_(t) is the tentative set of data subcarriers available to the user or region, and $\hat{\Gamma}_{u,k} \left( {\mathrm{t}} \right)$ is the predicted SINR of user *u* on subcarrier *k*. The summation converts the predicted per-tone SINRs into the ranking-rate quantity.

32 $$\overline{R}_{u} \left( t \right) = 0.9\overline{R}_{u} \left( {t - 1} \right) + 0.1R_{u} \left( t \right),\quad R_{u} \left( t \right) = 0\;{\mathrm{if}}\;{\mathrm{unscheduled}}$$

Equation (32) updates the throughput memory $\overline{R}_{u} \left( {\mathrm{t}} \right)$ from its previous value and the actually served aggregate rate *R*_*u*_(t). The coefficients 0.9 and 0.1 give a ten-frame-scale exponential memory, and *R*_*u*_(t) is set to zero when the user is not scheduled. Equation (33) then defines the OMN-PF ranking score *M*_*u*_(t).

33 $$\begin{gathered} M_{u} \left( t \right) = \frac{{\hat{R}_{u} \left( t \right)}}{{\left( {\overline{R}_{u} \left( t \right) + \varepsilon } \right)^{0.20} }}\left[ {1 + 2{\mathrm{max}}\left( {O_{u} ,O_{{{\mathrm{mem}},u}} } \right) + 14E_{u} + 0.5B_{u} } \right. \hfill \\ \left. {\quad \quad \quad \quad + 8\left\{ {0.75F_{u}^{{{\mathrm{NLOS}}}} O_{u} + 0.25\left( {1 - F_{u}^{{{\mathrm{NLOS}}}} } \right)\left( {1 - O_{u} } \right)} \right\} + 6S_{u} } \right] \hfill \\ \end{gathered}$$

where ε prevents division by zero and the exponent α = 0.20 reduces the dominance of the historical-rate denominator relative to conventional PF. *E*_*u*_(t) is one for an edge user and zero otherwise; *B*_*u*_(t), *F*_*u*_^NLOS^(t), and *S*_*u*_(t) are the transition-risk, NLOS-fragility, and service-gap terms defined above. The coefficients (2, 14, 0.5, 8, 6) weight outage risk, edge status, transition risk, the NLOS/outage interaction, and service gap. The interaction 0.75*F*_*u*_^NLOS^*O*_*u*_ + 0.25(1 − *F*_*u*_^NLOS^)(1 − *O*_*u*_) gives its largest emphasis when NLOS dependence and outage risk are simultaneously high, while retaining a smaller stabilizing contribution for strong LOS links. Conventional PF uses $\hat{R}_{u} /(\overline{R}_{u} + \varepsilon )$ with exponent one and no reliability multiplier^46^. OMN-PF and OMN-PF-A use the same ranking and differ only in post-admission allocation.

### Variable multi-user OFDMA admission

Users are ordered within each logical region and admitted sequentially. Equation (34) gives the resource-only admission ceiling for a center or edge pool before the link-quality and regional-rate tests are applied.

34 $$K_{p} \left( t \right) = {\mathrm{floor}}\left( {\frac{{N_{p} \left( t \right)}}{{N_{{{\mathrm{min}}}} }}} \right)$$

In Eq. (34), *K*_*p*_(t) is the maximum number of users that pool *p* can support under the minimum-share rule, *N*_*p*_(t) is the number of data subcarriers in that pool, and *N*_*min*_ = 16 is the minimum effective allocation per admitted user. With *N*_*c*_ = 113 and *N*_*e*_ = 47, the corresponding ceilings are seven center users and two edge users. The first ranked user is admitted so that an active region is not left idle solely because its link is weak; later candidates must satisfy Eq. (35).

35 $$n_{u} \ge N_{{{\mathrm{min}}}} ,\;\;\hat{\gamma}_{v} \ge \gamma_{{{\mathrm{adm}}}} \;\;\forall v \in {\mathcal{A}}_{b} \left( t \right),\;\;\mathop \sum \limits_{v} R_{v}^{{{\mathrm{tent}}}} \ge \beta_{R} R_{b,1}$$

In Eq. (35), *n*_*u*_ is the tentative number of subcarriers assigned to user *u*; $\hat{\gamma}_{v}$ is the predicted post-sharing SINR of tentative user *v*; *A*_*b*_(t) is the tentative admitted set in region *b*; *R*_*v*_^tent^ is a tentative user rate; and *R*_*b*,1_ is the first-user regional sum-rate reference. The thresholds are *γ*_*adm*_ = 10 dB and *β*_*R*_ = 0.55. These common feasibility controls are fixed heuristic settings, not optimized constants or a stochastic traffic model, and they are applied identically to RR1, PF, OMN-PF, and OMN-PF-A.

### OMN-PF and OMN-PF-A resource-allocation modes

After admission, OMN-PF divides the active center or edge pool equally among admitted users and allocates regional power in proportion to their tone counts, giving an approximately uniform power-per-subcarrier baseline. OMN-PF-A retains the same fixed δ = 2/3 partition, ranking, and admission rule, but introduces bounded adaptive tone and power weights.

Equation (36) converts the previously defined reliability states into the adaptive OFDMA weight *w*_*u*_^sc^. The clipping operator limits this weight to [0.75, 4.0], preventing extreme differences in tone allocation.

36 $$w_{u}^{{{\mathrm{sc}}}} = {\mathrm{clip}}_{{[0.{75},{ 4}.0]}} \left( {{1} + {1}.{5}0\,{\mathrm{O}}_{u} + {1}.{25}\,{\mathrm{E}}_{u} + {\mathrm{S}}_{u} + 0.{35}\,{\mathrm{B}}_{u} + 0.{35}\,{\mathrm{F}}_{u}^{{{\mathrm{NLOS}}}} {\mathrm{O}}_{u} } \right)$$

In Eq. (37), *n*_*u*_ is the integer tone count assigned to user *u*, *N*_*p*_ is the size of the current center or edge pool, and the denominator normalizes the adaptive weights over all admitted users *v* in that pool. The operator round*int*(·) denotes deterministic integer rounding followed by a correction step so that Σ*n*_*u*_ = *N*_*p*_ exactly.

37 $$n_{u} = {\mathrm{round}}_{{{\mathrm{int}}}} \left[ {{\mathrm{N}}_{p} {\mathrm{w}}_{u}^{{{\mathrm{sc}}}} /\sum\limits_{{v \in Ab({\mathrm{t}})}} {{\mathrm{w}}_{v}^{{{\mathrm{sc}}}} } } \right],\;\;\sum\limits_{{u \in Ab({\mathrm{t}})}} {{\mathrm{n}}_{u} = {\mathrm{N}}_{p} }$$

Equation (38) defines two bounded power-redistribution terms. The channel tilt *c*_*u*_ is clip[0.75, 1.50](√(*H*_*ref*_/*H*_*u*_)), where *H*_*ref*_ is the median positive channel gain of the admitted users in the active region and *H*_*u*_ is the current gain of user *u*. The reliability tilt *r*_*u*_ increases with outage risk, waiting time, and edge status but is capped at 1.75. The bounds prevent either term from creating an extreme power imbalance.

38 $$c_{u} = {\mathrm{clip}}_{{\left[ {0.75,1.50} \right]}} \sqrt {\frac{{H_{{{\mathrm{ref}}}} }}{{H_{u} }}} ,\;\;r_{u} = {\mathrm{min}}\left\{ {1.75,1 + 0.35O_{u} + 0.25S_{u} + 0.25E_{u} } \right\}$$

Equation (39) normalizes the user weights to preserve the regional power budget. Here *P*_*b*_ is the fixed power budget of active region *b*, *P*_*u*_ is the power assigned to user *u*, and *n*_*u*_*c*_*u*_*r*_*u*_ is the user’s combined tone-count, channel, and reliability weight. The denominator sums this weight over all admitted users *v* in the region, so Σ*P*_*u*_ = *P*_*b*_ up to floating-point precision.

39 $$P_{u} = P_{b} \frac{{n_{u} c_{u} r_{u} }}{{\mathop \sum \nolimits_{v} n_{v} c_{v} r_{v} }}$$

The adaptive formulas are bounded heuristics rather than a globally optimal joint admission, OFDMA, and power solution. They redistribute a fixed resource and power budget; they do not add transmit power, change the strict-FFR edge pools, or adapt δ, which remains fixed at 2/3.

### Algorithmic implementation

Algorithm 3 summarizes the source-truth dynamic experiment: state updates, policy-specific ranking, common sequential admission, OMN-PF or OMN-PF-A post-admission allocation, and all-user service accounting.

**Algorithm 3:**
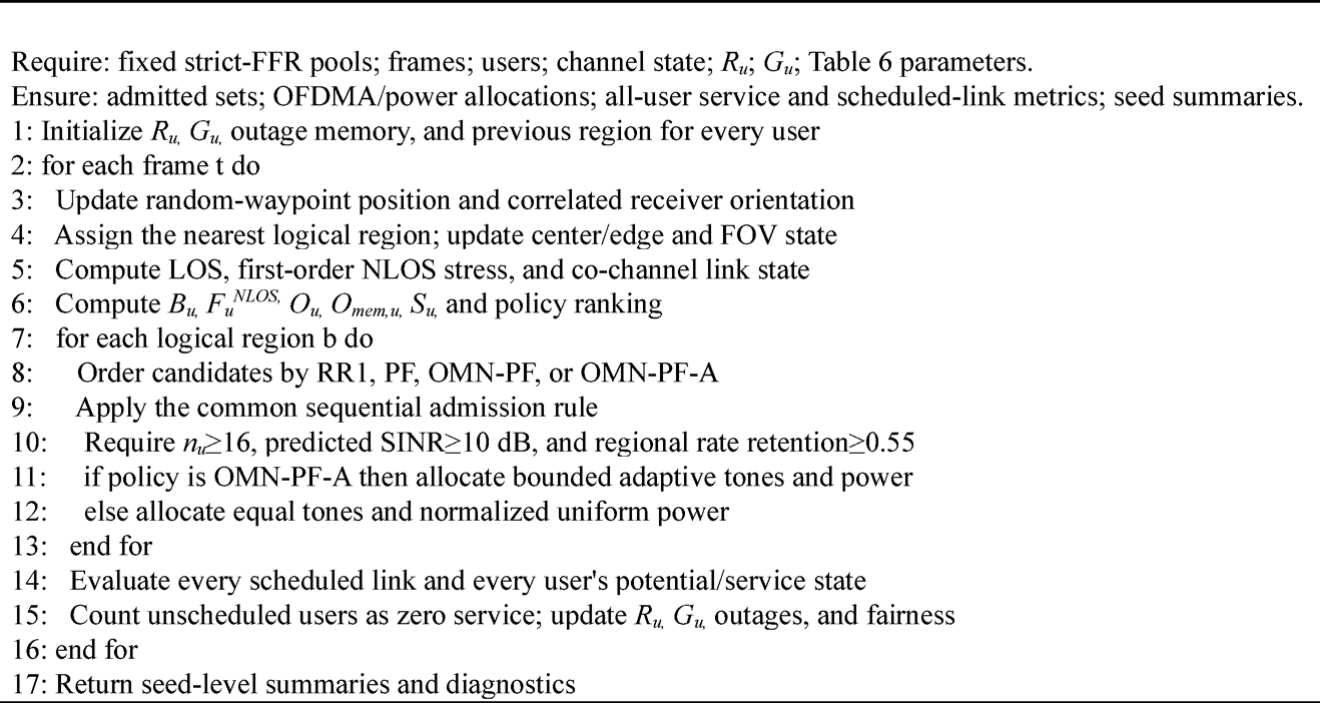
Source-truth dynamic OMN-PF/OMN-PF-A experiment

#### Dynamic simulation setup and metrics

Table 6 reports the dynamic source-truth geometry, mobility, orientation, reflection-stress, ranking, memory, admission, and adaptive-allocation parameters. Ten independent seeds are used; Table 7 reports seed means and 95% confidence half-widths.

**Table 7:** Dynamic comparison under mobility, correlated orientation, and first-order NLOS stress. Entries are mean ± seed-level 95% confidence half-width over 10 independent seeds (300 trials/seed, 75 frames/trial).

Scheduler	Edge C10 (%)	Edge C20 (%)	Scheduled aggregate SE	Mean per-frame Jain index	Mean 20-dB outage duration (frames)
RR1	3.76 ± 0.05	1.71 ± 0.03	78.65 ± 0.36	0.0435 ± 0.0001	38.22 ± 0.13
PF	8.25 ± 0.05	3.23 ± 0.03	144.19 ± 0.55	0.0855 ± 0.0004	31.54 ± 0.13
OMN-PF	11.37 ± 0.05	4.97 ± 0.04	159.25 ± 0.66	0.0859 ± 0.0003	32.67 ± 0.15
OMN-PF-A	11.56 ± 0.05	6.11 ± 0.05	159.18 ± 0.63	0.0866 ± 0.0003	31.07 ± 0.16

On a Dell Precision Tower 5810 equipped with an Intel Xeon E5-2660 v3 processor (10 cores, 20 threads, 2.60 GHz) and 32 GB RAM, the complete experiment remained an overnight-scale computation using four parallel workers. The final run covered 10 independent seeds, 300 trials per seed, and 75 frames per trial for RR1, PF, OMN-PF, OMN-PF-A, and the additional reference/ablation configurations in the code package. The mean recorded simulator time was approximately 1,347 s per seed for OMN-PF and 2,564 s per seed for OMN-PF-A, so the adaptive mode required about 90% more computation under the same workload. These values are hardware- and implementation-dependent and are reported only as indicative reproducibility costs.

For each eligible user-trial pair, Edge Cγ is the number of edge-classified frames in which that user is scheduled and its scheduled-link SINR is at least γ, divided by its number of edge-classified frames. An unscheduled edge frame therefore counts as failure. Average scheduled aggregate SE is conditional on admitted links. Jain’s index is computed each frame over all 42 user rates, including zeros, then averaged. Outage duration is the mean length of consecutive 20 dB service-outage episodes, where non-scheduling or sub-threshold service counts as outage.

#### Dynamic results and interpretation

**Fig. 11:**
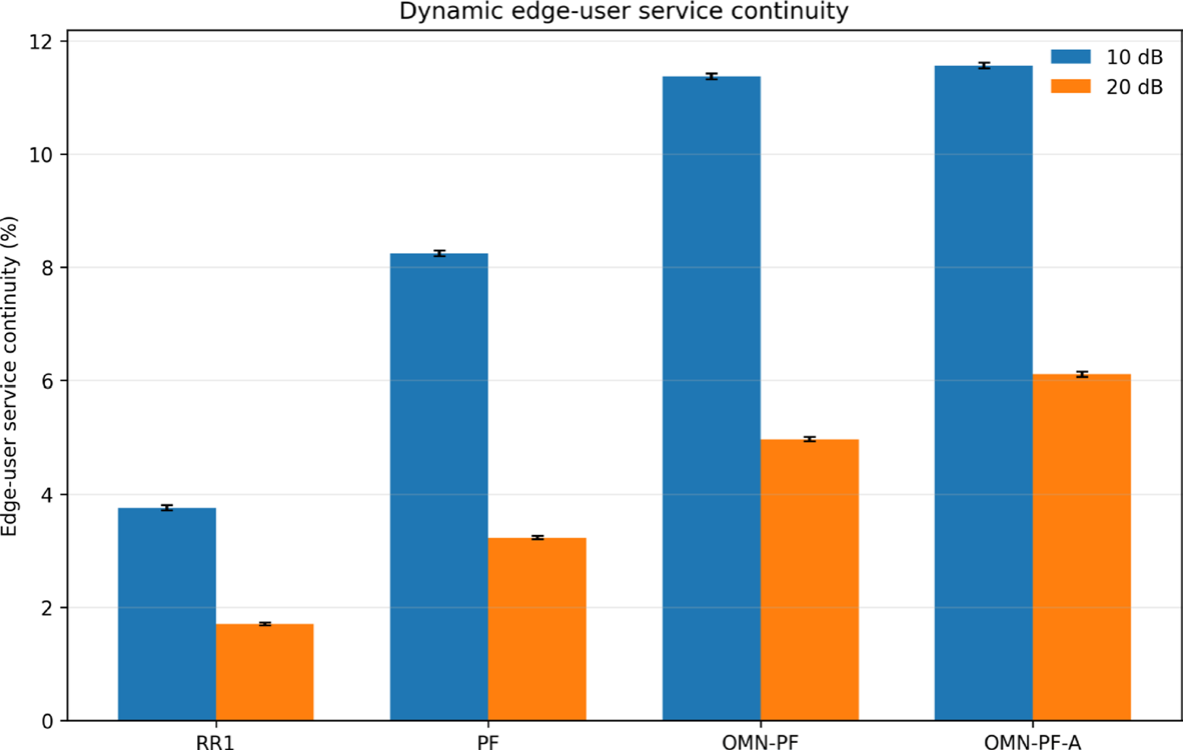
All-edge-frame service success at 10 and 20 dB. Unscheduled edge frames count as unsuccessful; error bars are seed-level 95% confidence intervals.

**Fig. 12:**
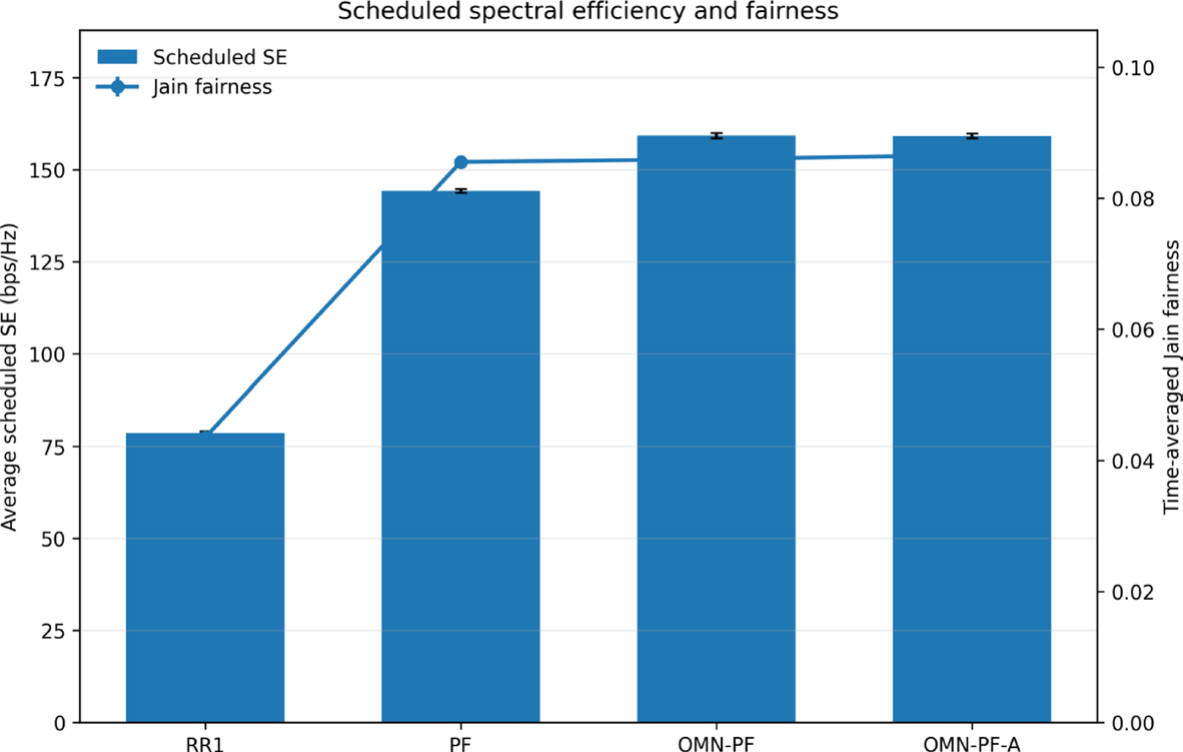
Scheduled-user aggregate SE (conditional on admission) and mean per-frame Jain index over all user rates, including zeros. Error bars are seed-level 95% confidence intervals.

PF, OMN-PF, and OMN-PF-A achieve absolute 20 dB edge-frame service success of 3.23%, 4.97%, and 6.11%. OMN-PF’s relative improvements over PF are 37.9% at 10 dB and 54.0% at 20 dB, while conditional scheduled aggregate SE rises from 144.19 to 159.25. The low absolute success rates show that all policies remain weak under the adopted stress profile (Figs. 11 and 12).

OMN-PF-A increases 20 dB edge-frame success from 4.97% to 6.11% relative to OMN-PF while conditional scheduled aggregate SE is statistically similar (159.18 versus 159.25). Mean per-frame Jain indices remain low: 0.0859 and 0.0866. Relative gains do not imply unconditional continuity or deployment readiness.

OMN-PF and OMN-PF-A admit about 1.20 users per active region on average and preserve the regional power budget to numerical precision. These implementation diagnostics do not measure packet latency, packet error rate, energy efficiency, or signaling overhead.

The dynamic results are simulation stress-test estimates. Calibrated ray tracing, measured obstruction, hardware nonlinearities, synchronization error, delayed/noisy CSI, packet-level handover, distributed coordination, multi-room scalability, and external AI/global-optimization baselines remain untested.

## Limitations and scope of validity

This is a simulation-based benchmark and stress extension, not a hardware-validated deployment study. Static rr1 metrics are conditional sampled-link statistics; dynamic scheduled aggregate SE is conditional on admission, whereas edge-frame success and per-frame Jain fairness count unscheduled service appropriately.

The static channel is LOS-only. The dynamic first-order reflection term uses deliberate stress multipliers and coarse patches, not calibrated ray tracing, measured reflectance, furniture, or human-body blockage.

Mobility and correlated orientation alter association and service, but lower-layer handover signaling, packet buffering/loss, authentication, and make-before-break latency are not simulated.

All schedulers use centralized simulated state. Distributed synchronization, CSI acquisition and delay, inter-AP signaling, control-channel cost, and implementation latency remain outside scope.

The static power/bias routine omits finite LED headroom and residual clipping; the dynamic allocator is heuristic rather than globally optimal.

## Conclusion

The static study provides a source-audited ADT-SDMA-OFDMA strict-FFR benchmark. The canonical archive gives mean SINR near 28 dB, but the rr1 aggregate-SE and Jain values are conditional sampled-link statistics. The controlled no-FFR variant lowers mean SINR by about 8 dB; angular-relaxation variants are compound TX/RX changes and are not causal reproductions of prior systems.

In the dynamic stress experiment, OMN-PF and OMN-PF-A improve relative edge-frame service success over PF, but absolute 20 dB values remain only 4.97% and 6.11% and per-frame Jain indices remain below 0.087. These modes are bounded heuristic trade-offs, not universally dominant schedulers. Hardware validation, calibrated propagation, packet-level metrics, distributed coordination, scalability, and external optimization/AI benchmarks remain future work.

## Supplementary Information

Below is the link to the electronic supplementary material.

Supplementary Material 1

## Data Availability

The data generated and analysed during the current study are available in two Zenodo repositories. The simulation outputs, source code, and reproducibility materials for the static ADT-SDMA-OFDMA strict-FFR baseline are archived at 10.5281/zenodo.19714290, with the corresponding development repository available at https://github.com/Mostfa3385/lifi-adt-ofdma-ffr. The simulation outputs, source code, and reproducibility materials for the dynamic mobility, NLOS, and OMN-PF extension are archived at 10.5281/zenodo.21309355, with the corresponding development repository available at https://github.com/Mostfa3385/lifi-omn-pf-dynamic. These repositories contain the files used to generate the figures, tables, and reported numerical results.

## Code availability

The custom Python simulation code and computational pipeline used in this study are deposited in recognized DOI-assigning repositories. The static baseline version-of-record archive is available on Zenodo at 10.5281/zenodo.19714290 (release v1.0.2), and the accompanying development repository is available on GitHub at https://github.com/Mostfa3385/lifi-adt-ofdma-ffr. The static archive contains the simulator (simulator.py), experiment drivers (run_experiments.py, run_orientation_sweep.py, run_comparison.py), the implementation corresponding to Algorithms 1 and 2, configuration settings, CSV result files, and README instructions for reproducing the Monte Carlo tables, sensitivity sweeps, ablation comparison, and figures. The dynamic extension code and result files for mobility, receiver-orientation variation, first-order NLOS stress, and service-gap-aware OMN-PF scheduling are archived on Zenodo at 10.5281/zenodo.21309355 (release v1.1.1; Service-Gap-Aware OMN-PF Scheduling for Dynamic ADT-OFDMA Strict-FFR LiFi Attocell Networks; Service-gap-aware OMN-PF dynamic simulator v1.1.1), and the accompanying GitHub repository is available at https://github.com/Mostfa3385/lifi-omn-pf-dynamic. Zenodo is the persistent archival record; the GitHub repositories are provided for transparency and development history.
